# Harnessing the reverse cholesterol transport pathway to favor differentiation of monocyte-derived APCs and antitumor responses

**DOI:** 10.1038/s41419-023-05620-7

**Published:** 2023-02-15

**Authors:** Laura Raccosta, Maura Marinozzi, Susan Costantini, Daniela Maggioni, Lorena Maria Ferreira, Gianfranca Corna, Paola Zordan, Angela Sorice, Diego Farinello, Silvia Bianchessi, Michela Riba, Dejan Lazarevic, Paolo Provero, Matthias Mack, Attilio Bondanza, Ivan Nalvarte, J-A Gustafsson, Valeria Ranzani, Francesco De Sanctis, Stefano Ugel, Silvère Baron, Jean-Marc A. Lobaccaro, Lorenzo Pontini, Manuela Pacciarini, Catia Traversari, Massimiliano Pagani, Vincenzo Bronte, Giovanni Sitia, Per Antonson, Andrea Brendolan, Alfredo Budillon, Vincenzo Russo

**Affiliations:** 1 Immuno-Biotherapy of Melanoma and Solid Tumors Unit, Division of Experimental Oncology, IRCCS Scientific Institute San Raffaele, Milan, 20132 Italy; 2 Big Ideas in Organic Synthesis (BIOS) Laboratory, Department of Pharmaceutical Sciences, University of Perugia, Perugia, 06123 Italy; 3 Experimental Pharmacology Unit, Laboratori di Mercogliano, Istituto Nazionale Tumori IRCCS Fondazione G. Pascale, Naples, Italy; 4 Division of Immunology, Transplantation and Infectious Diseases, IRCCS San Raffaele Scientific Institute, 20132 Milan, Italy; 5 Lymphoid Organ Development Unit, Division of Experimental Oncology, IRCCS Scientific Institute San Raffaele, Milan, 20132 Italy; 6 Center for Translational Genomics and Bioinformatics IRCCS Scientific Institute San Raffaele, Milan, 20132 Italy; 7 Division of Internal Medicine II-Nephrology, University of Regensburg, Regensburg, 93042 Germany; 8 Innovative Immunotherapy Unit, IRCCS Scientific Institute San Raffaele, Milan, 20132 Italy; 9 Department of Biosciences and Nutrition, Karolinska Institutet, Huddinge, S-14183 Sweden; 10 Center for Nuclear Receptors and Cell Signaling, Department of Biology and Biochemistry, University of Houston, Houston, TX 77004 USA; 11 Istituto Nazionale Genetica Molecolare Romeo ed Enrica Invernizzi, 20122 Milan, Italy; 12 Department of Medicine, Section of Immunology, Verona University Hospital, 37134 Verona, Italy; 13 Université Clermont Auvergne, GReD, CNRS, INSERM, and Centre de Recherche en Nutrition Humaine d’Auvergne Clermont-Ferrand, Clermont-Ferrand, France; 14 MolMed S.p.A., Milan, 20132 Italy; 15 Department of Medical Biotechnology and Translational Medicine, Università degli Studi di Milano, 20133 Milan, Italy; 16 Veneto Institute of Oncology - Istituto di Ricovero e Cura a Carattere Scientifico (IOV-IRCCS), 35128 Padova, Italy; 17 Vita-Salute San Raffaele University, 20132 Milan, Italy

**Keywords:** Immunoediting, Immune evasion, Cancer microenvironment, Antigen-presenting cells, Immunotherapy

## Abstract

Lipid and cholesterol metabolism play a crucial role in tumor cell behavior and in shaping the tumor microenvironment. In particular, enzymatic and non-enzymatic cholesterol metabolism, and derived metabolites control dendritic cell (DC) functions, ultimately impacting tumor antigen presentation within and outside the tumor mass, dampening tumor immunity and immunotherapeutic attempts. The mechanisms accounting for such events remain largely to be defined. Here we perturbed (oxy)sterol metabolism genetically and pharmacologically and analyzed the tumor lipidome landscape in relation to the tumor-infiltrating immune cells. We report that perturbing the lipidome of tumor microenvironment by the expression of sulfotransferase 2B1b crucial in cholesterol and oxysterol sulfate synthesis, favored intratumoral representation of monocyte-derived antigen-presenting cells, including monocyte-DCs. We also found that treating mice with a newly developed antagonist of the oxysterol receptors Liver X Receptors (LXRs), promoted intratumoral monocyte-DC differentiation, delayed tumor growth and synergized with anti-PD-1 immunotherapy and adoptive T cell therapy. Of note, looking at LXR/cholesterol gene signature in melanoma patients treated with anti-PD-1-based immunotherapy predicted diverse clinical outcomes. Indeed, patients whose tumors were poorly infiltrated by monocytes/macrophages expressing LXR target genes showed improved survival over the course of therapy. Thus, our data support a role for (oxy)sterol metabolism in shaping monocyte-to-DC differentiation, and in tumor antigen presentation critical for responsiveness to immunotherapy. The identification of a new LXR antagonist opens new treatment avenues for cancer patients.

## Introduction

Immune checkpoint inhibitors (ICIs) counteracting inhibitory signals of T cell activation have revolutionized the antitumor treatments and improved the overall survival of patients affected by different tumor types [1]. However, still many patients do not respond or progress after an initial benefit [2]. ICI-resistant patients often display signatures of poor antigen presentation. Poor antigen presentation can be associated to the reduced density of dendritic cells (DCs) in the tumor microenvironment (TME) [3], to the dampening of DC antigen-presenting abilities [4] or the inhibition of DC differentiation and maturation [3]. Restoring DC functions or recruiting alternative sources of antigen-presenting cells remains crucial for non-responding patients with defects of antigen-presenting cells.

Many products from cholesterol and lipid metabolism exert immunosuppression by dampening the stimulatory functions of dendritic cells (DCs) [5]. Different mechanisms can be responsible for the accumulation of lipids in DCs, i.e. the up-regulation of receptors promoting lipid uptake or mechanisms involving the endoplasmic reticulum stress response via the transcription factor X-box-binding protein 1 (XBP1) [6]. Oxidized cholesterol metabolites, namely oxysterols, were shown to modulate DC functions by inhibiting the expression of the chemokine receptor CCR7, thus inhibiting their migration to draining lymphnodes and tumor immunity [7]. Oxysterols can be inactivated by the enzyme sulfotransferase 2B1b (SULT2B1b), which generates sulfated oxysterols [8]. SULT2B1b has been shown to impair Liver X Receptor (LXRs) signaling [9, 10] in response to dietary cholesterol [11], contribute to T cell proliferation after TCR engagement [12], generate intrinsic regulators of thymocyte development [13], and restore antitumor immune responses when over-expressed by tumor cells, through the modulation of the migratory ability of tumor-infiltrating dendritic cells (DCs) [7] and neutrophils [14]. Whether SULT2B1b and sulfated oxysterols regulate other cellular processes during tumor formation, such as myeloid cell differentiation remains unknown.

Monocytes are circulating phagocytic mononuclear cells capable of differentiating into either tissue macrophages or in potent antigen presenting cells (i.e. monocyte-derived DCs) [15, 16]. In established tumors, classic Ly6C^+^ monocytes migrate to tumors in a C-C chemokine receptor-2 (CCR2)-dependent manner and mainly differentiate into tumor-associated macrophages, which contribute to tumor progression, metastasis formation and therapy resistance [17]. While this differentiation pathway is well established, very little is known on the route leading to the intratumor differentiation of Ly6C^high^ monocytes towards monocyte-DCs (Mono-DCs). Anthracycline-induced cell death was recently shown to promote the recruitment and differentiation/activation of monocyte-derived DCs into the TME, and these cells cross-presented TAAs to CD8^+^ T cells [18]. Recently, the activation of the transcription factor p53 induced by inflammation, was shown to differentiate Ly6C^+^CD103^+^ monocytic antigen-presenting cells in tumors [19]. Whether other pathways, such as metabolic pathways, contribute to monocyte monocyte-DC differentiation remains poorly known.

Here, we show that SULT2B1b system manipulation favors the differentiation of Ly6C^+^ monocytes into monocyte-DCs. This pathway is associated with lipidome reprogramming characterized by reduced levels of cellular cholesterol/cholesterol derivatives and glycerophospholipids, and increased levels of polyunsaturated fatty acid precursors of lipid mediators endowed with pro-inflammatory activity [20]. This lipidomic profile was also detected in tumors from mice treated with the new synthetic compound PFM037, structurally and functionally related to SULT2B1b-derived products. PFM037 contributed to monocyte differentiation and induced effective antitumor responses by interfering with LXR signaling. Moreover, it increased the efficacy of immune checkpoint blockers and adoptive cell therapy. Finally, by interrogating available scRNA-seq datasets of tumor-infiltrating immune cells, we reported a significant enrichment of melanoma-infiltrating monocytes/macrophages over-expressing the LXR target genes *ABCA1* and *SCD1* in ICB-resistant patients.

## Results

### The oxysterol-inactivating enzyme SULT2B1b promotes intratumor accumulation of CD3^+^CD8^+^ T-cells and monocyte-derived antigen-presenting cells (APCs)

**Fig. 1:**
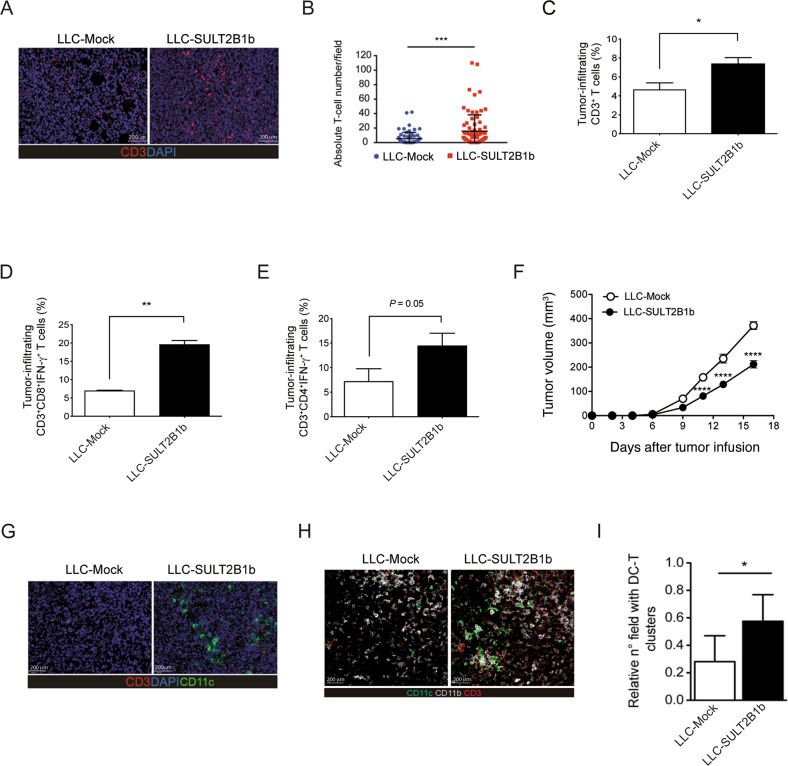
Analysis of the LLC tumor microenvironment under SULT2B1b perturbation. **A** One representative confocal microscopy imaging of CD3^+^ T cells infiltrating LLC-Mock and LLC-SULT2B1b tumors. Red, CD3^+^ T cells; Blue, DAPI (bars = 200 µm). **B** Absolute T cell number/field evaluated by counting 4 different experiments with FIJI software (extension of ImageJ) and classical cell counter. DAPI^+^ events (blue) displaying CD3 staining (red) were considered as positive T cells. ***P < 0.001 (Student’s t-test). **C** Flow cytometry analysis of the percentage of CD45^+^CD3^+^ T cells infiltrating LLC-Mock and LLC-SULT2B1b tumors. *P < 0.05 (Student’s t-test). Mean and s.d. of four experiments. **D** Flow cytometry analysis of the percentage of CD3^+^CD8^+^INF-γ^+^ T cells infiltrating LLC-Mock and LLC-SULT2B1b tumors. **P < 0.01 (Student’s t-test). Mean and s.d. of two experiments. **E** Flow cytometry analysis of the percentage of CD3^+^CD4^+^INF-γ^+^ T cells infiltrating LLC^-^Mock and LLC-SULT2B1b tumors. *P* = 0.01 (Student’s t-test). Mean and s.d. of two experiments. (**F**) LLC-Mock and LLC-SULT2B1b tumor growth. Mean and s.d. of one experiment (*n* = 10 mice/group). ****P < 0.0001 (Student’s t-test). (**G** and **H**) Representative confocal microscopy imaging of CD3^+^ T cells and CD11c^+^ cells (**G**) and CD3^+^ T cells, CD11b^+^ and CD11c^+^ cells (**H**) infiltrating LLC-Mock and LLC-SULT2B1b tumors. Red, CD3^+^ T cells; Blue, DAPI, Green, CD11c; Gray, CD11b (bars = 200 µm). (**I**) Quantification of the fields of DC/T cell clusters evaluated by counting 4 different experiments with FIJI software (extension of ImageJ) and classical cell counter. The number of sections in each image displaying the presence of both DCs (CD11c^+^CD11b^+^) and T cells (CD3^+^) was considered as a positive DC/T cell cluster. *P < 0.05 (Student’s t-test).

**Fig. 2:**
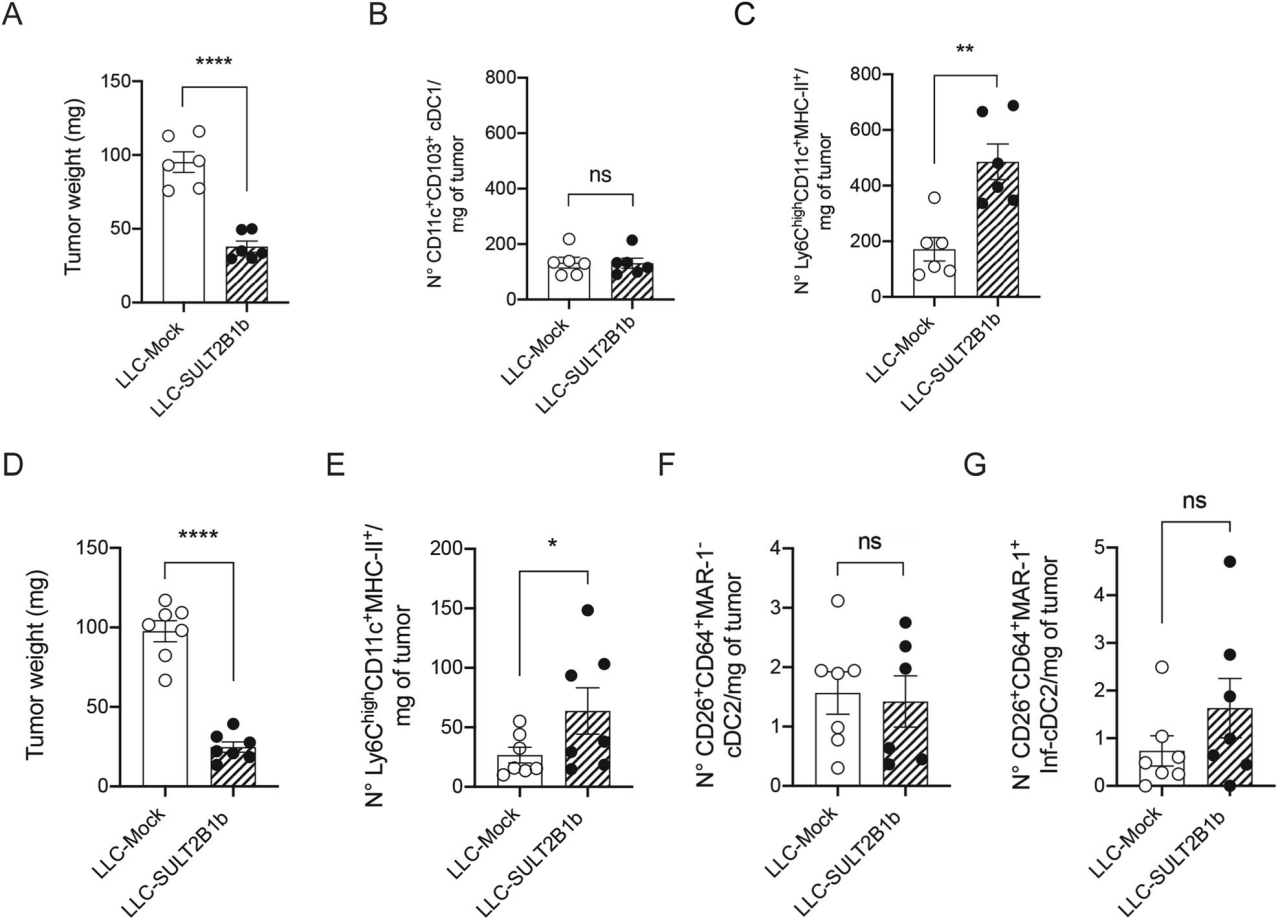
Analysis of the DC subsets infiltrating LLC tumor microenvironment under SULT2B1b perturbation. **A** Mice challenged with LLC-Mock and LLC-SULT2B1b tumors were sacrificed 12 days after tumor challenge and evaluated for tumor weight. Mean and s.d. of 6 mice/group. ****P < 0.0001 (Student’s t-test). The same tumors were analyzed for the number of CD11c^+^CD103^+^ cDC1 (**B**) and monocyte-DCs/mg of tumors (**C**). Mean and s.d. of 6 mice/group. **P < 0.01; ns, not significant (Student’s t-test). **D** Mice challenged with LLC-Mock and LLC-SULT2B1b tumors were sacrificed 10 days after tumor challenge and evaluated for tumor weight. Mean and s.d. of 7 mice/group. ****P < 0.0001 (Student’s t-test). **E**-**G** Numbers of monocyte-DCs (**E**) CD26^+^CD64^+^MAR-1^-^ cDC2 (**F**) and CD26^+^CD64^+^MAR^-^1^+^ Inf-cDC2/mg of tumors (**G**)^.^ Mean and s.d. of 6-7 mice/group. *P < 0.05 (Student’s t-test).

We analyzed the microenvironment of the mouse Lewis Lung Carcinoma (LLC) expressing or not SULT2B1b and observed an increase in CD3^+^ T cells infiltrating LLC-SULT2B1b tumors (Fig. 1A-C). We detected higher percentages of CD8^+^IFN-γ^+^ and CD4^+^IFN-γ^+^ T cells (Fig. 1D, E), which paralleled a better control of tumor growth in mice bearing LLC-SULT2B1b tumors (Fig. 1F). Of note, LLC-SULT2B1b and LLC-Mock grew in a similar manner in vitro, and in vivo in NOD-SCID mice (Suppelementary Fig. 1A and [7]). Thus, indicating that tumor growth control is mediated by the activity of SULT2B1b on immune cells infiltrating tumors. Since the increased number of T-cells frequently associates with high numbers of tumor-infiltrating DCs [21, 22], we evaluated the presence and number of CD11c^+^ cells infiltrating SULT2B1b- and Mock-LLC tumors by confocal microscopy analysis. We detected higher numbers of both CD11c^+^CD11b^+^ cells (Fig. 1G) and DC-T cell clusters in LLC-SULT2B1b tumors (Fig. 1H, I). To better define the subsets of DCs infiltrating SULT2B1b- and Mock-LLC tumors, we performed extensive FACS analysis. Twelve days after tumor challenge, we sacrificed tumor-bearing mice and analyzed tumor weights (Fig. 2A) and tumor-infiltrating DCs (Fig. 2B, C), the latter based on the expression of CD45, CD11c, CD11b, CD103, Ly6C and MHC-II markers. We did not observe different numbers of classic CD11c^+^CD103^+^ cDC1 between SULT2B1b- and Mock-LLC tumors (Fig. 2B). Instead, under SULT2B1b perturbation we observed increased numbers of Ly6C^high^CD11c^+^MHC-II^+^ cells (Fig. 2C and Supplementary Fig. 1B, C), resembling monocyte-derived DCs as suggested by the expression of CD11b, Sirp-α, Sca-1, CCR2, CD209a and PD-L2 markers (Supplementary Fig. 1D) [16, 23]. The intratumor number of these monocyte-derived DCs (hereafter referred as mono-DCs) increased over time and correlated with a better control of tumor growth (Supplementary Fig. 2A). We also observed the increase in a heterogeneous population of CD11b^+^Ly6C^low^ cells (hereafter referred as Ly6C^low^ cells) infiltrating LLC-SULT2B1b tumors, some of them expressing CD11c and MHC-II molecules and resembling inflammatory monocytes and/or macrophages (Supplementary Fig. 2B). Functionally, FACS-purified mono-DCs elicited OVA-specific naïve OT-I and OT-II T cells more efficiently than Ly6C^low^ cells (Supplementary Fig. 2C-E), suggesting a primary role played by mono-DCs in eliciting effective antitumor immune responses in our conditions.

**Fig. 3:**
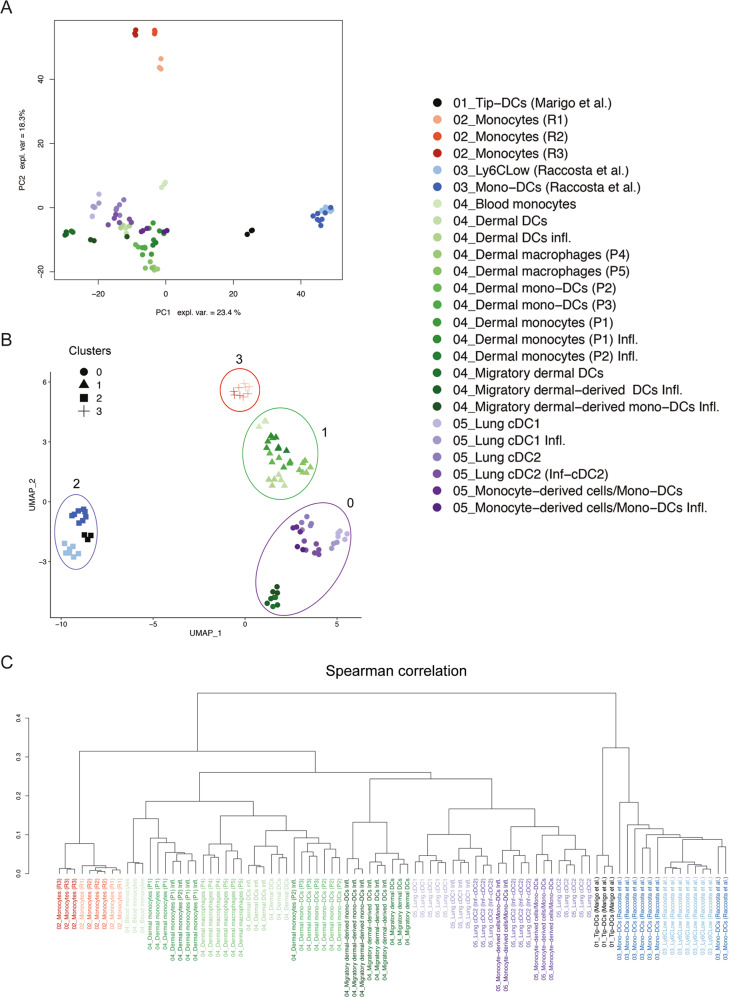
Transcriptomic features of monocyte-derived cells under SULT2B1b perturbation. **A** Principal Component Analysis (PCA) of the transcriptomes of mono-DCs and Ly6C^low^ cells isolated from LLC-Mock and LLC-SULT2B1b tumors, integrated with 4 datasets present in the GEO (Gene Expression Omnibus) Database. PCA based on the expression values of 10080 genes detected in all the five experimental datasets. The first two principal components are shown (PC1 accounted for 23.4 % and PC2 for 18.3% of the variance among samples). **B** Uniform Manifold Approximation and Projection analysis (UMAP) of the transcriptomes as reported in (**A**). The figure shows bidimensional UMAP representation of the integrated dataset. Four different clusters have been identified using graph-based techniques. **C** Hierarchical clustering analysis. Hierarchical clustering of the expression values of 10080 genes detected with average linkage was performed on the specified subsets to assess their transcriptional relatedness. Spearman correlation-based distance was used.

Because mono-DCs share similar phenotypes as the inflammatory cDC2 (inf-cDC2) [24], we analyzed and compared cDC2 and Inf-cDC2 in SULT2B1b- and Mock-LLC-bearing mice, based on the expression of CD45, CD11c, CD11b, Ly6C, CD26, CD64, MAR-1 and MHC-II markers (Supplementary Fig. 2F-H). As previously shown, tumor weights (Fig. 2D) and numbers of mono-DCs (Fig. 2E) were lower and higher in LLC-SULT2B1b tumors, respectively. The numbers of tumor-infiltrating CD26^+^CD64^+^MAR-1^-^ cDC2 and CD26^+^CD64^+^MAR-1^+^ inf-cDC2 [24] were instead very low and similar between LLC-Mock and LLC-SULT2B1b (Fig. 2F, G). These results prompted us to perform RNA-seq experiments of mono-DCs and Ly6C^low^ cells isolated from SULT2B1b- and Mock-LLC tumors to obtain deeper molecular clues (Supplementary Fig. 3A, B). Ly6C^high^CD11c^+^MHC-II^+^ mono-DCs from both Mock- and SULT2B1b-tumors expressed transcripts peculiar of mono-DCs, such as *Cd209a*, *Nos2, C5ar1*, *Cx3cr1* and *FcγRI* (Supplementary Fig. 3C) [23, 25]. Of note, Ly6C^low^ cells isolated from SULT2B1b-LLC tumors expressed higher levels of transcripts encoding MHC-II, CD74 and CIITA molecules (Supplementary Fig. 3B). Both subsets (i.e. monocyte-DCs and Ly6C^low^ cells) isolated from SULT2B1b-LLC tumors, expressed high levels of *Cxcl9* and *Cxcl10* transcripts, which encode chemokines involved in the recruitment of antitumor effector T cells [26] (Supplementary Fig. 3D, E) and lower levels of the *Cebpb* transcripts, whose increased expression has been associated with the immune suppressive abilities of bone marrow-derived, tumor-infiltrating myeloid cells [27] (Supplementary Fig. 3F, G). The transcription factor *Irf8* was expressed at high levels by both subsets isolated from Mock- and SULT2B1b-LLC tumors, whereas *Irf4*, which regulates monocyte-DC differentiation [28], was expressed at higher levels in mono-DCs isolated from SULT2B1b-LLC tumors as compared to cells isolated from Mock-LLC (Supplementary Fig. 3F). Then, we compared our transcriptomic results with publicly available datasets, particularly TNF- and NOS-producing DCs (Tip-DCs) [25], bone marrow-derived and blood monocytes [23], skin-derived macrophages, cDCs and monocyte-DCs [29], and lung-derived cDC1, cDC2, inf-cDC2 and monocyte-derived APCs [24]. Ly6C^high^CD11c^+^MHC-II^+^ mono-DCs, as well as Ly6C^low^ cells isolated from SULT2B1b- and Mock-LLC tumors resulted to be closer to the Tip-DCs (Fig. 3A). By UMAP analyses we identified 4 clusters grouping cells isolated from different organs or pathologic conditions sharing some program commonalities (Fig. 3B). Cluster 2 grouped our mono-DCs and Ly6C^low^ cells with Tip-DCs [25], both groups isolated from tumors. Cluster 0, cells isolated from lung independently of their state of activation [24]. Cluster 0 also encompassed DC and monocytes isolated from lymph nodes. Cluster 1 grouped cells isolated from skin independently of their state of activation [29], and Cluster 3 monocytes isolated from blood or bone marrow [23]. These results, also confirmed by the hierarchical clustering analysis (Fig. 3C), indicate that cell identities account for the tissue or the pathologic condition where the cells come from (Fig. 3B), as previously demonstrated for macrophages isolated from different organs [30, 31].

To get deeper insights into the pathways modulated by SULT2B1b-perturbed mono-DCs and Ly6C^low^ cells, we performed gene set enrichment analyses (GSEA) of the transcriptomic data by Hallmarks, which highlighted the enrichment of specific metabolic pathways (Supplementary Fig. 4A, and Supplementary Tables 1 and 2). Oxidative phosphorylation, adipogenesis, fatty acid metabolism and cholesterol metabolism were significantly up-regulated in mono-DCs from SULT2B1b-LLC tumors (Supplementary Fig. 4A). Cholesterol and fatty acid pathways were also up-regulated in Ly6C^low^ cells (Supplementary Fig. 4B). The up-regulation of cholesterol pathway in cells from LLC-SULT2B1b tumors was also confirmed using a previously reported cholesterol synthesis gene signature [32] (Supplementary Fig. 4C). We also observed the down-regulation of the LXR target gene *Abca1* [33] in the cells isolated from LLC-SULT2B1b tumors (Supplementary Fig. 4D), in accordance with previous in vivo data [11].

Altogether, these results indicate that under SULT2B1b manipulation there is an intratumor increase of activated monocyte-derived cells correlating with a better control of tumor growth.

### Under SULT2B1b perturbation tumor-recruited monocytes differentiate into DCs

The above-reported results suggest that in certain conditions, i.e. under SULT2B1b perturbation, the immunostimulatory ability of monocyte-derived DCs can be exploited to induce effective antitumor responses. To validate this hypothesis, we performed tumor challenge experiments by systemically depleting CCR2^+^ monocytes, using the monoclonal antibody MC-21 [34], or by blocking the egress of monocytes from the bone marrow towards inflammatory sites taking advantage of CCR2-deficient mice [35], under SULT2B1b manipulation. Mice bearing SULT2B1b-tumors treated with an anti-CCR2 depleting mAb [34] for five consecutive days were infiltrated by lower numbers of mono-DCs, identified by the expression of Ly6C, CD11b and Sca-1 markers, and failed to control tumor growth (Supplementary Fig. 5A, B). In accordance with these results, *Ccr2*^*-/-*^ mice failed to control SULT2B1b-tumors as compared to *wild type* mice (Supplementary Fig. 5C). Ly6C^high^CD11c^+^MHC-II^+^ mono-DCs were virtually absent in SULT2B1b-tumors grown in *Ccr2*^*-/-*^ mice (Supplementary Fig. 5D). Ly6C^low^CD11c^+^MHC-II^+^ cells were lower in SULT2B1b-tumors grown in *Ccr2*^*-/-*^ mice as compared to *wild type* mice (Supplementary Fig. 5E). The number of CD11c^+^CD103^+^ cDCs were not significantly different among the group of tumors grown in *Ccr2*^*-/-*^ and *wild type* mice (Supplementary Fig. 5F, G). These experiments suggest that increasing the intratumor number of monocyte-derived antigen-presenting cells improves the antitumor immune responses.

**Fig. 4:**
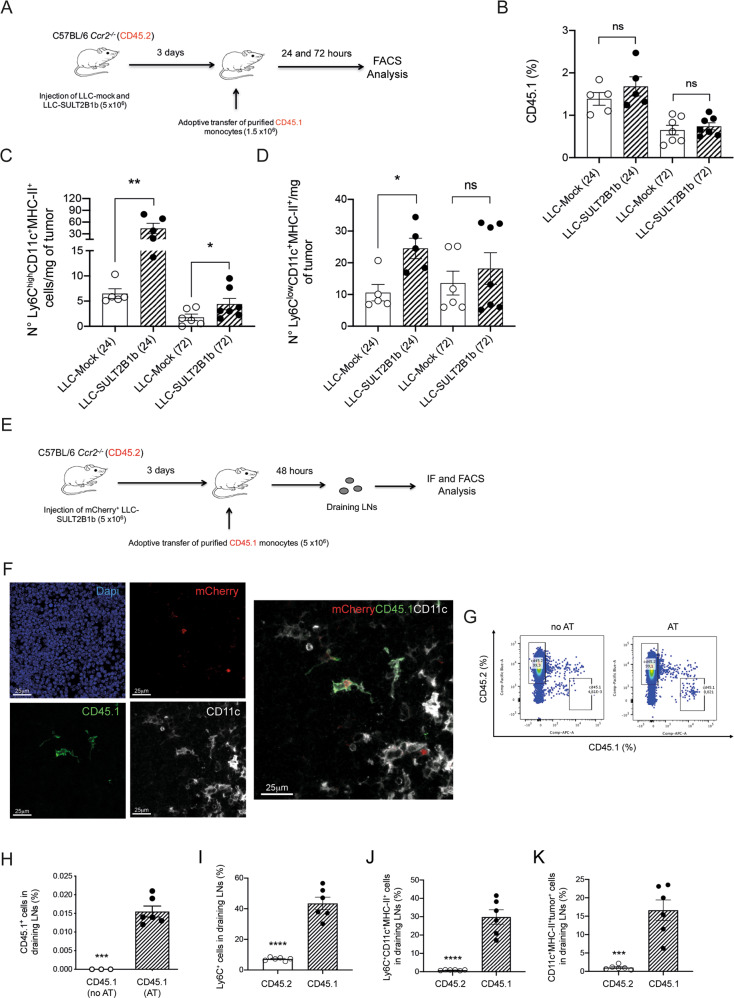
SULT2B1b expression promotes intratumor differentiation of monocyte-DCs migrating to draining lymph nodes. **A** Schematic for assessing intratumor monocyte-DC differentiation. CD45.2^+^*Ccr2*^*-/-*^ mice bearing LLC-Mock and LLC-SULT2B1b tumors were administered with CD45.1^+^ bone-marrow purified monocytes (Ly6C^+^). Twenty-four hours later tumors were collected and analyzed by flow cytometry. **B** Percentage of CD45.1^+^ cells infiltrating LLC-Mock and LLC-SULT2B1b tumors 24 and 72 hours after the adoptive transfer of pure CD45.1^+^Ly6C^+^ monocytes. Mean and s.d. of 5 (24 h) and 7 (72 h) mice/group. No difference was observed between the two groups. ns, not significant. **C**-**D** Numbers of mono-DCs (**C**) and Ly6C^low^CD11c^+^MHC-II^+^ cells (**D**) infiltrating LLC-Mock and LLC-SULT2B1b tumors 24 and 72 hours after the adoptive transfer of pure CD45.1^+^Ly6C^+^ monocytes. Mean and s.d. of 5 (24 h) and 6-7 (72 h) mice/group. ns, not significant; *P < 0.05; **P < 0.01 (Student’s t-test). **E** Schematic for assessing tumor-engulfed monocyte-DCs in the draining lymph nodes. CD45.2^+^*Ccr2*^*-/-*^ mice bearing LLC-SULT2B1b tumors expressing mCherry were administered with CD45.1^+^ bone-marrow purified monocytes (Ly6C^+^). Forty-eight hours later draining lymph nodes were collected and analyzed by flow cytometry. As control, draining lymph nodes were collected from not adoptively transferred tumor-bearing mice. **F** Representative confocal microscopy imaging of LLC-SULT2B1b tumors expressing mCherry^+^, CD45.1^+^, CD11c^+^ cells in the draining lymph nodes. Red, mCherry^+^; Blue, DAPI, Green, CD45.1; Gray, CD11c (bars = 25 µm). **G** Representative flow cytometry image of CD45.1^+^ and CD45.2^+^ cells in the draining lymph nodes of tumor-bearing mice. (**H**) Quantification of CD45.1^+^ cells in the draining lymph nodes of tumor-bearing mice with or without adoptive transfer of CD45.1^+^ monocytes. Mean and s.d. of 3 (no AT) and 6 (AT) mice/group. ***P < 0.001 (Student’s t-test). **I**-**K** Quantification of CD45.1^+^Ly6C^+^ or CD45.2^+^Ly6C^+^ cells (**I**), Ly6C^+^CD11c^+^MHC^-^II^+^ cells (**J**) and CD11c^+^MHC^-^II^+^mCherry^+^ cells (**K**) in the draining lymph nodes of tumor-bearing mice adoptively transferred with bone marrow-derived CD45.1^+^ monocytes. The CD11c^+^MHC-II^+^mCherry^+^ cells are tumor^-^engulfed mono-DCs migrated to draining lymph nodes. Mean and s.d. of 6 mice/group. ***P < 0.001; ****P < 0.0001 (Student’s t-test).

Then, we asked whether the increased numbers of mono-DCs in SULT2B1b-tumors were due either to an enhanced recruitment of monocytes or to their intratumor differentiation. To address this issue, we performed cell lineage tracing experiments by using congenic CD45 mice (Fig. 4A). CD45.2^+^ *Ccr2*^*-/-*^ mice bearing 3-day-established SULT2B1b- or Mock-LLC tumors were injected with bone marrow-purified CD45.1^+^Ly6C^+^ monocytes. One and three days later, tumors were collected and analyzed for the content of mono-DCs and Ly6C^low^CD11c^+^MHC-II^+^ cells. The percentage of CD45.1^+^ cells recruited to SULT2B1b- and Mock-LLC tumors was similar (Fig. 4B), while the number of mono-DCs was higher in LLC-SULT2B1b both at 24 and 72 hours (Fig. 4C). The number of Ly6C^low^CD11c^+^MHC-II^+^ cells was higher only at 24 hours (Fig. 4D). At 72 hours the number of intratumor mono-DCs was greatly reduced, suggesting their possible migration to draining lymph nodes. These experiments indicate that under SULT2B1b perturbation, there is an increased rate of monocytes undergoing monocyte-to-DC differentiation. Based upon the results obtained at 72 hours, we analyzed whether mono-DCs generated in SULT2B1b-perturbed tumors were able to migrate to draining lymph nodes, where antitumor immune responses occur. *Ccr2*^*-/-*^ CD45.2^+^ mice were challenged with SULT2B1b-LLC expressing the mCherry fluorescent protein, and five days later with CD45.1^+^ purified monocytes (Fig. 4E). Two days later we collected and analyzed by confocal microscopy draining lymph nodes, and visualized CD11c^+^CD45.1^+^ cells phagocytosing mCherry^+^ bodies (Fig. 4F). By FACS analysis of the cell suspension of draining lymph nodes we provided evidence and quantified the percentage of CD45.1^+^ cells (Fig. 4G, H), also expressing Ly6C, CD11c and MHC-II molecules (Fig. 4I, J). Approximately 16% of CD45.1^+^Ly6C^+^CD11c^+^MHC-II^+^ cells were mCherry^+^, indicating that these cells were able to phagocytose tumor-associated proteins and migrate to draining lymph nodes (Fig. 4K), whereby directly [18] or indirectly [36] activate effective antitumor responses.

### Sulfated oxysterols promote LXR-dependent monocyte-DC differentiation and antitumor responses

**Fig. 5:**
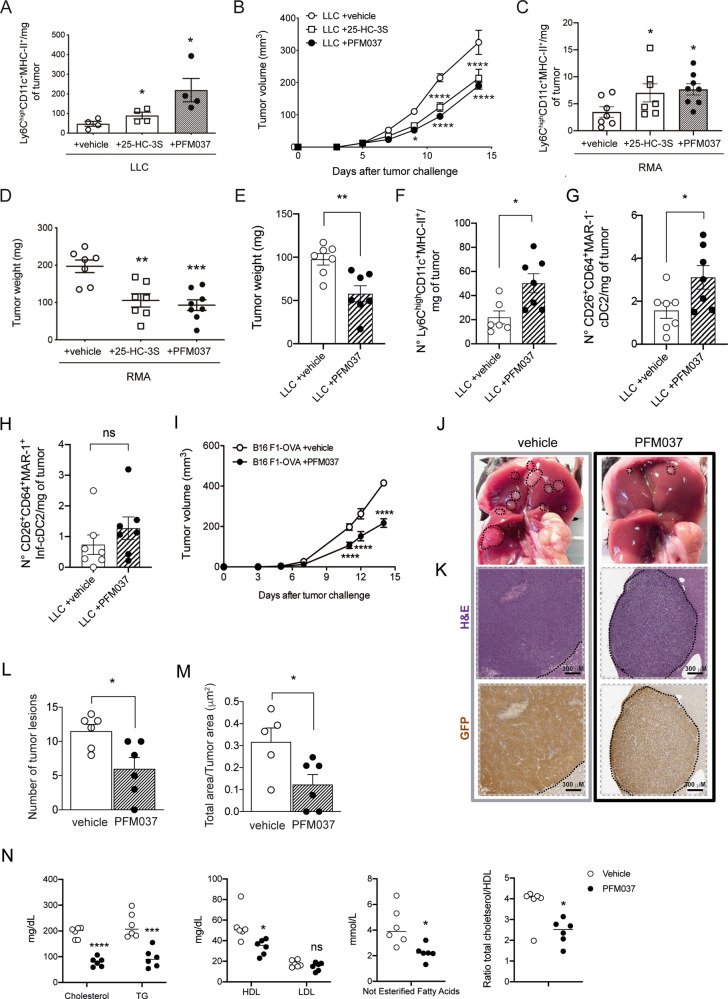
Sulfated oxysterols promote monocyte-DC differentiation and antitumor activity. **A** Mice bearing established LLC tumors were treated with the sulfated oxysterols 25-HC-3S, PFM037 or vehicle. At sacrifice, absolute numbers of Ly6C^high^CD11c^+^MHC-II^+^ monocyte-DCs/mg of LLC tumors, were evaluated. Mean and s.d. of 4 mice/group. *P < 0.05 (Anova). **B** LLC-bearing mice were treated with the sulfated oxysterols 25-HC-3S, PFM037 or vehicle and monitored for tumor growth. Mean and s.d. of one experiment (*n* = 6 mice/group). *P < 0.05; ****P < 0.0001 (Anova). **C** Mice-bearing established RMA tumors were treated with the sulfated oxysterol 25-HC-3S, PFM037 or vehicle. At sacrifice, absolute numbers of Ly6C^high^CD11c^+^MHC-II^+^ monocyte-DCs/mg of RMA tumors, were evaluated. Mean and s.d. of 7-8 mice/group. *P < 0.05 (Anova). **D** RMA-bearing mice were treated with the sulfated oxysterols 25-HC-3S, PFM037 or vehicle and evaluated for tumor weight at mice sacrifice. Mean and s.d. of 7-8 mice/group. **P < 0.01; ***P < 0.001 (Anova). **E**-**H** Tumor weights (**E**) and numbers of mono-DCs (**F**), cDC2 identified by the expression of CD26 and CD64 markers (**G**), and Inf-DC2 identified by the expression of CD26, CD64 and MAR-1 markers (**H**) infiltraing tumors from mice treated with vehicle or PFM037. Mean and s.d. of 6-7 mice/group. ns, not significant; *P < 0.05; **P < 0.01 (Student’s t-test). The difference of the numbers of mono-DCs between the experiments reported in **A** and **F** is due to the different days of analysis after tumor challenge and treatment, 12 and 10 days in A and F, respectively. **I** Melanoma B16F1-OVA growth delay following the treatment with PFM037. B16F1-OVA-bearing mice were treated with vehicle or with PFM037. Mean and s.d. of one experiment (*n* = 8 mice/group). **P < 0.01; ***P < 0.001; ****P < 0.0001 (Student’s t-test). (**J**) Representative images of liver metastases 21 days after injection of CRC in mice treated with vehicle (gray frame) or PFM037 (black frame). The black dashed line indicates tumor lesions. (**K**) Representative H&E and GFP immunohistochemical micrographs showing liver tumor features of metastases found in vehicle- (gray frame) or PFM037-treated (black frame) mice 21 days after intramesenteric injection of MC38-GFP cells. Scale bar = 300μm. **L**, **M** Quantification of the number of macroscopic liver tumor lesions (**L**), as well as the percentage of tumor area (**M**) determined by IHC in the mice described in **J**-**K**. Mean values ± s.d. are shown; *P < 0.05 (Mann-Whitney test). **N** Plasma levels of total cholesterol, triglycerides (TG), high and low-density lipoproteins (HDL, LDL), and high-density lipoproteins (HDL), Not Esterified Fatty Acids, and in vehicle and ratio between total cholesterol and HDL in vehicle and PFM037 (0.4 μg/day)-treated mice. *P < 0.05; ***P < 0.001; ****P < 0.0001 (Anova).

We hypothesized that sulfated sterols, which are generated by SULT2B1b [8], could condition the differentiation of tumor-recruited monocytes. To investigate this hypothesis, we evaluated whether the administration of sulfated oxysterols could induce antitumor responses. Tumor-bearing mice were treated with the commercially available 25-hydroxycholesterol-3 sulfate (25-HC-3S) or with a newly developed 3-sulfate form of the LXR-inactive oxysterol 22S-hydroxycholesterol (22S-HC), namely PFM037, obtained by chemical sulfation of the 22S-HC. Treatments started 6-7 days after tumor challenge, when tumors are well established. We detected higher absolute numbers of tumor-infiltrating mono-DCs (Fig. 5A), as well as the delay of tumor growth (Fig. 5B) in both 25-HC-3S- and PFM037-treated LLC-bearing mice. Of note, LLC tumor cells treated in vitro with 25-HC-3S, PFM037 or vehicle grew in a similar manner (Supplementary Fig. 1A). Higher numbers of tumor-infiltrating mono-DCs (Fig. 5C) and tumor growth control (Fig. 5D) were also observed when we treated mice bearing the RMA lymphoma with 25-HC-3S or PFM037. Yet in this experimental setting, the absolute number of cDC2 and Inf-DC2 were respectively 15 and 30 times lower than mono-DCs (Fig. 5E-H). PFM037 treatment also delayed the growth of the melanoma B16F1-OVA (Fig. 5I), indicating that different tumor types could benefit from therapy based on sulfate oxysterol administration. Finally, we tested PFM037 in a model of liver metastasis formation following intramesenteric injection of the MC38-GFP colon cancer cells [37, 38]. Seven days after tumor challenge mice were treated with PFM037 or vehicle 3 days/week for 2 weeks. Twenty-one days after tumor challenge, mice were sacrificed, and liver analyzed (Fig. 5J, K). Mice treated with PFM037 developed much less tumor nodules than vehicle-treated mice (Fig. 5L). In addition, the tumor area was reduced in PFM037-treated mice as compared to vehicle-treated mice (Fig. 5M). Of note, tumor-bearing mice treated with PFM037 showed reduced levels of plasma total cholesterol, triglycerides, not esterified fatty acids and HDL, whereas LDL did not change (Fig. 5N). Altogether, these findings indicate that sulfate oxysterols exogenously provided favor in vivo mono-DC differentiation and contribute to antitumor responses. Furthermore, these findings suggest a shared mechanism of mono-DC differentiation by SULT2B1b, 25-HC-3S and PFM037.

**Fig. 6:**
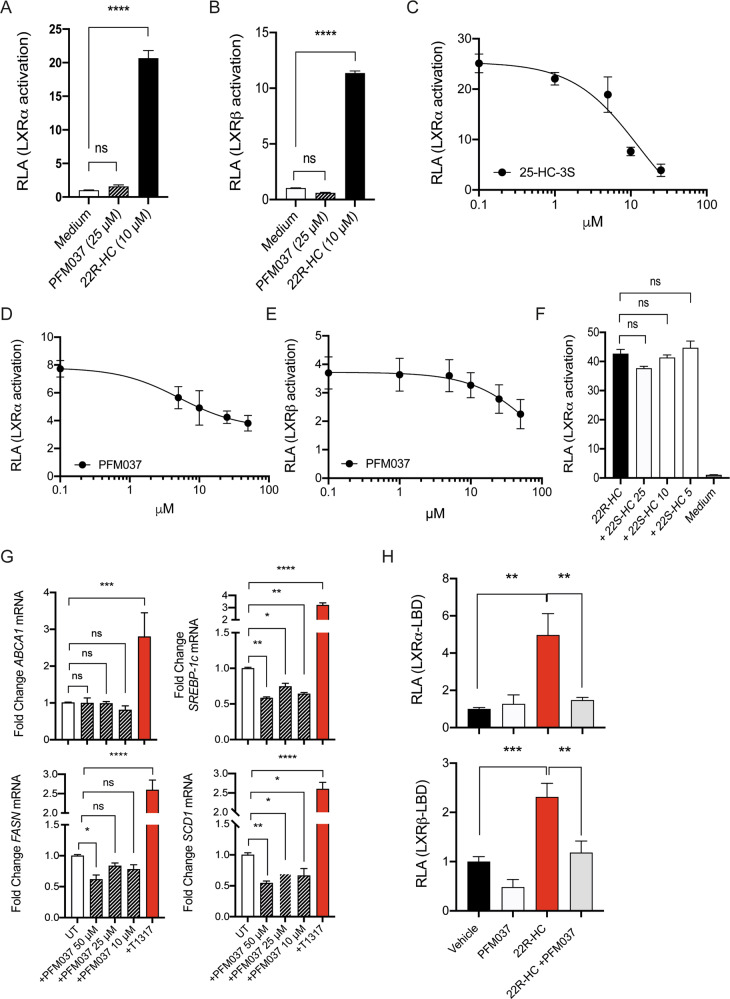
PFM037 interferes with LXR activity. **A**, **B** Analysis of luciferase-based LXRα (**A**) and LXRβ (**B**) activation induced by PFM037, or 22R-HC used as control. Mean and s.d. of three independent experiments. ****P < 0.0001 (Anova). (**C**-**D**) Luciferase-based LXRα assays displaying the antagonistic activity of 25-HC-3S (**C**) and PFM037 (**D**). Mean and s.d. of 2 independent experiments. LXRα activation was induced by 5 (**C**) or 10 μM (**D**) of 22R-HC, 25-HC-3S was used at 25, 10, 5 and 1 μM, whereas PFM037 at 50, 25, 10, 5 and 1 μM. RLA, Relative Luciferase Activity. **E** Luciferase-based LXRβ assays displaying the antagonistic activity of PFM037. Mean and s.d. of 2 experiments. LXRβ activation was induced by 10 μM of 22R-HC, PFM037 was used at 50, 25, 10, 5 and 1 μM. RLA, Relative Luciferase Activity. **F** Luciferase-based LXRα assays to test the antagonistic activity of the inactive oxysterol 22S-HC. 22S-HC does not antagonize LXR activation induced by 5 μM of 22R-HC. 22S-HC was used at 25, 10, 5 and 1 μM. RLA, Relative Luciferase Activity. **G** Analysis of the expression of the LXR target genes ABCA1, SREBP1c, FASN and SCD1 following T0901317 (T1317) or PFM037 treatment. Mean and s.d. of three independent experiments. *P < 0.05; **P < 0.01; ***P < 0.001; ****P < 0.0001 (Anova). **H** Mammalian two-hybrid assay in Huh7 cells using Gal4-DBD-tagged RAP250-Del4 (containing NR-box1), together with pVP16-LXRα-LBD or pVP16-LXRβ-LBD, and the reporter plasmid pUAS-tk-Luc. Values shown are the mean and s.d. of two experiments performed in triplicate with luciferase activity normalized to protein content and with the activity of respective pVP16-fusion without ligand set to 1.0. The ligands 22R-HC and PFM037 was used at 10 µM when indicated. **P < 0.01; ***P < 0.001 (Anova).

It has been shown that SULT2B1b/SULT2B1b-derived products antagonize LXR activity [7, 8, 11]. Therefore, we asked whether PFM037 was interfering with LXR activation/signaling. To address this issue, we set up different molecular assays. First, by luciferase-based reporter assays we demonstrated that PFM037, the new 3-sulfate form of 22S-HC obtained by its chemical sulfation, did not activate LXRα or β (Fig. 6A, B). Then, we tested the antagonism of LXRα or β activation induced by 22R-HC by using titrating amounts of 25-HC-3S, 22S-HC and PFM037 (Fig. 6C-F). 25-HC-3S and PFM037 antagonized the LXRα activity in a dose-dependent manner (Fig. 6C, D) with IC_50_ of 12.65 and 5.18 μM, respectively. PFM037 also antagonized LXRβ activity with an IC_50_ of 82.46 μM (Fig. 6E). Of note, 22S-HC did not antagonize LXR activity (Fig. 6F). Then, we tested by qPCR the capacity of PFM037 to activate the expression of the LXR target genes *ABCA1*, *SREBP1c*, *FASN* and *SCD1* in the U937 myeloid cells differentiated with PMA for 72 hours and then treated with T0901317 (10 μM) or with different concentrations of PFM037. As expected, we did not find any activation of *ABCA1* transcripts (Fig. 6G). Unexpectedly, we observed the inhibition of *SREBP-1c*, *FASN*, and *SCD1* mRNA expression (Fig. 6G). These results prompted us to test PFM037 by using the mammalian two-hybrid system and we took advantage of the Del4 construct, which is from the NR coactivator RAP250/NcoA6 containing the first NR-box that is known to bind to agonist-bound nuclear receptors [39]. Cells were transfected with Del4 plasmid together with either LXRα or LXRβ LBDs fused to the activating domain of VP16 [40]. The addition of 22R-HC induced luciferase activity, while the addition of PFM037 slightly inhibited the luciferase activity for LXRβ, thus suggesting that PFM037 antagonizes endogenous LXR ligands (Fig. 6H). In accordance with the luciferase assays reported above (Fig. 6C, D), PFM037 reduced the luciferase activity induced by 22R-HC (Fig. 6H). Altogether these results demonstrate that PFM037 is endowed with an LXR antagonistic activity. Moreover, the reduction of luciferase activity in the absence of agonists (Fig. 6H), as well as the reduction of *SREBP1c*, *FASN* and *SCD1* transcripts, suggest that PFM037 may behave as an inverse agonist of LXRs.

**Fig. 7:**
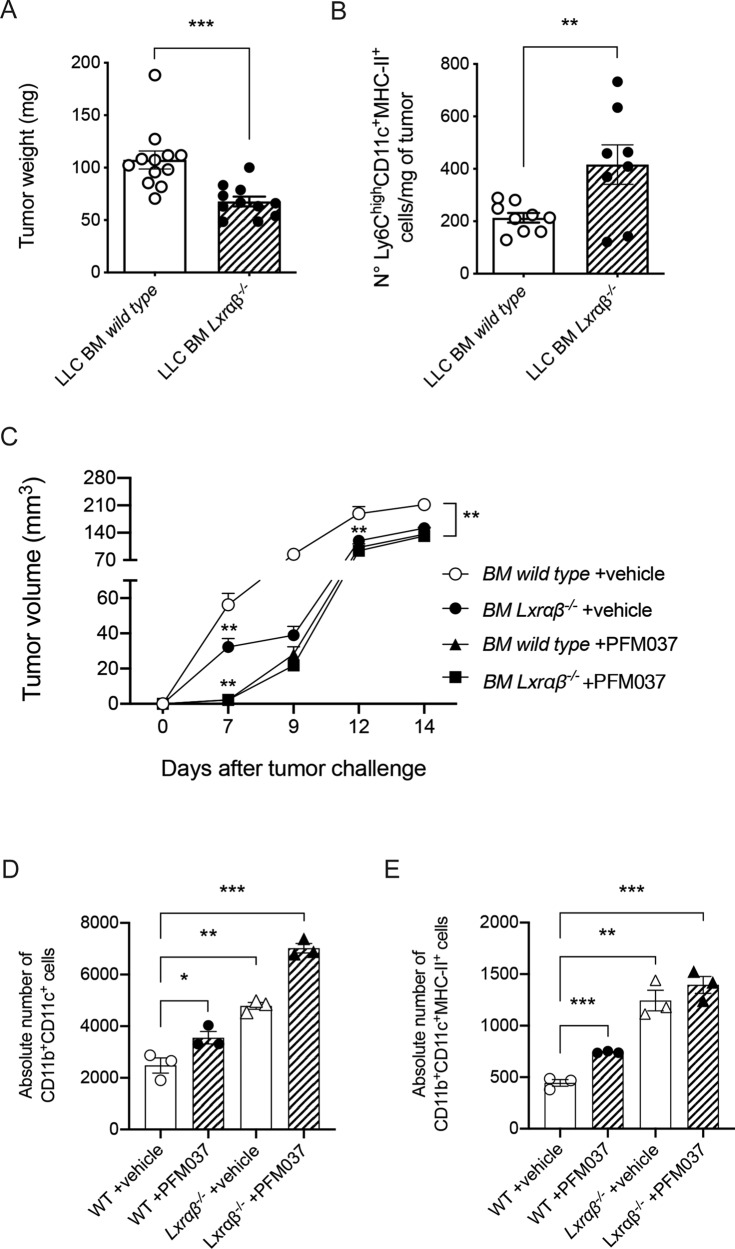
LXR signaling contributes to in vitro and in vivo PFM037-induced monocyte-to-DC differentiation. **A**, **B** Tumor weight (**A**) and numbers (**B**) of Ly6C^high^CD11c^+^MHC-II^+^ mono-DCs/mg of LLC-Mock tumors grown in *Lxrαβ*^*-/-*^ or wild type bone-marrow chimera mice. Mean and s.d. of 2 experiments (*n* = 11-12 mice/group). ***P < 0.001 (Student’s t-test) for tumor weight. Mean and s.d. of 2 experiments (*n* = 8 mice/group). **P < 0.01 (Student’s t-test) for Ly6C^high^CD11c^+^MHC-II^+^ mono-DC content. (**C**) LLC tumor growth in in *Lxrαβ*^*-/-*^ or wild type bone*-*marrow chimera mice treated with vehicle or PFM037. Mean and s.d. of one experiment (*n* = 5-7 mice/group). **P < 0.01 (Student’s t-test). (**D** and **E**) Absolute number of *wild type* or *Lxrαβ*^*-/-*^ monocytes expressing CD11b^*+*^CD11c^+^ (**D**) and CD11b^+^CD11c^+^MHC-II^+^ (**E**) after 48 hours of culture in the presence of vehicle or PFM037. Mean and s.d. of 3 independent experiments. *P < 0.05; **P < 0.01; ***P < 0.001 (Anova).

To investigate the involvement of LXR in monocyte activation as well as in monocyte-to-DC programming and antitumor responses, we took advantage of *Lxrαβ*^*-/-*^ mice. We challenged wild-type and *Lxrαβ*^*-/-*^ bone marrow (BM) chimera mice with LLC tumor cells. *Lxrαβ*^*-/-*^ BM-chimera mice controlled LLC tumor growth better than LLC-bearing wild-type chimera mice (Fig. 7A) and were infiltrated by a higher number of mono-DCs (Fig. 7B), suggesting a possible role exerted by LXR signaling in the differentiation of tumor-infiltrating monocytes. We performed similar experiments in *Lxrαβ*^*-/-*^ and wild-type BM-chimera mice challenged with LLC tumor cells and treated with vehicle or PFM037. We observed a better control of LLC growth by *Lxrαβ*^*-/-*^ BM-chimera mice treated with vehicle or PFM037 and by wild-type BM-chimera mice treated with PFM037 as compared to wild-type BM-chimera mice treated with vehicle (Fig. 7C). Moreover, early after tumor injection we noticed a significant difference between mice treated with PFM037 (both *wild type* and *Lxrαβ*^*-/-*^ BM chimera) and those treated with vehicle (Fig. 7C), suggesting the co-existence of LXR-dependent and -independent mechanisms, exerted by PFM037 at different times.

To investigate whether PFM037 promotes mono-DC differentiation in vitro, we cultured bone marrow-purified monocytes with M-CSF in the presence or in the absence of PFM037. Monocytes cultured with PFM037 expressed CD11c marker as early as after 48 hours of culture, with a smaller fraction of cells expressing MHC-II^+^ molecules (Fig. 7D, E). The involvement of LXR signaling was demonstrated by culturing *Lxrαβ*^*-/-*^ monocytes with M-CSF in the presence or in the absence of PFM037 (Fig. 7C, D). In this condition, we observed the up-regulation of CD11c (Fig. 7D) and MHC-II (Fig. 7E) molecules also in the absence of PFM037. Of note, the up-regulation of CD11c was even higher in *Lxrαβ*^*-/-*^ monocytes treated with PFM037 (Fig. 7D). Altogether, these experiments indicate that SULT2B1b/LXR axis contributes to monocyte differentiation towards mono-DCs, which in turn contribute to tumor growth control.

### Analysis of the lipidomic/metabolomic landscape of SULT2B1b-perturbed and PFM037-treated tumors

**Fig. 8:**
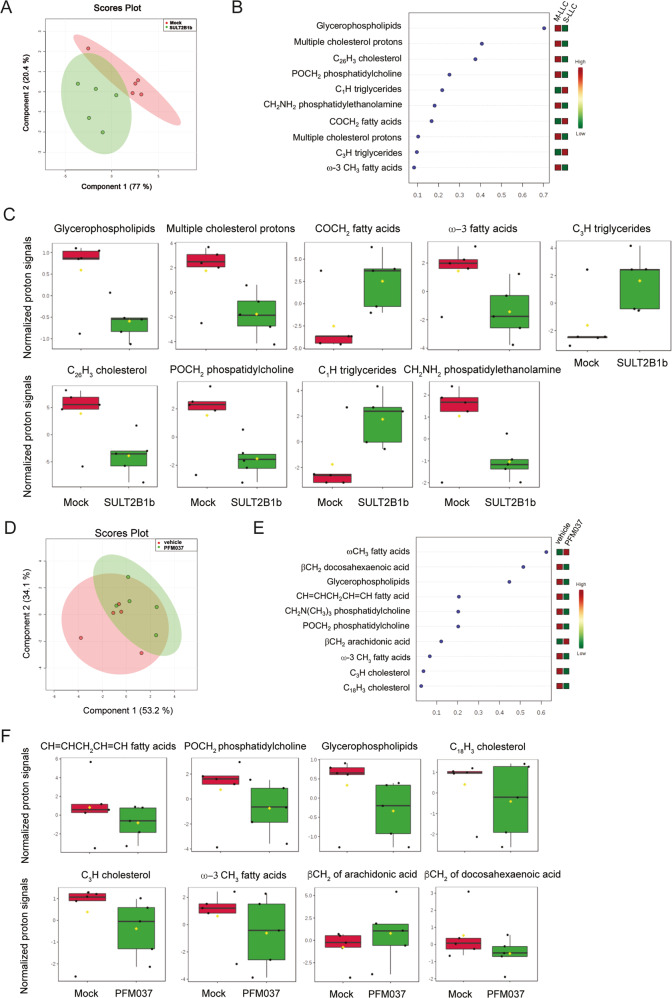
Lipidomic landscape of LLC tumors under SULT2B1b or PFM037 perturbation. **A** Score plot by partial least squares-discriminant analysis (PLS-DA) related to the lipidic fraction of Mock and SULT2B1b-LLC tumors (*n* = 5 tumors/group). **B** Variable importance in projection (VIP) plot showing the top 10 NMR signals resulted more statistically different in the lipidic fractions of Mock- and SULT2B1b-LLC tumors in C57Bl/6 mice (*n* = 5 tumors/group). **C** Box plots showing the levels of lipid species, evaluated as normalized proton signals, differentially expressed by Mock- and SULT2B1b-LLC tumors (*n* = 5 tumors/group). **D** Score plot by partial least squares-discriminant analysis (PLS-DA) related to the lipidic fraction of vehicle- and PFM037-treated-LLC tumors (*n* = 5 tumors/group). **E** Variable importance in projection (VIP) plot showing the top 10 NMR signals resulted more statistically different in the lipidic fractions of vehicle- and PFM037-treated-LLC tumors in C57Bl/6 mice (*n* = 5 tumors/group). **F** Box plots showing the levels of lipid species, evaluated as normalized proton signals, differentially expressed by LLC tumors from mice treated with vehicle of PFM037 (*n* = 5 tumors/group).

GSEA of SULT2B1b-perturbed mono-DCs (Supplementary Fig. 4) and the molecular analyses of PFM037 prompted us to carry out un-targeted metabolomics/lipidomics profiling of SULT2B1b- and PFM037-perturbed tumors by Nuclear Magnetic Resonance (NMR) spectroscopy, aimed to identify metabolic changes and commonalities between SULT2B1b- and PFM037-perturbed tumors. Mock- and SULT2B1b-LLC tumors grouped into two different clusters in the score plot, indicating the presence of significant different levels of lipidic species between the two groups. We observed a significant reduction in SULT2B1b-LLC tumors of proton signals related to cholesterol/cholesterol products, glycerophospholipids among them phosphatidylcholine and phosphatidylethanolamine, and polyunsaturated ω-3 fatty acids (Fig. 8A-C). Saturated fatty acids and triglycerides were instead enriched in SULT2B1b-LLC tumors (Fig. 8A-C). A similar profile was also observed in PFM037-treated tumors, as compared to vehicle-treated tumors (Fig. 8D-F). Notably, PFM037-treated tumors were enriched in proton signals of ω-6 fatty acid arachidonic acid and its derivatives, indicating an enrichment of lipids endowed with pro-inflammatory functions [20] (Fig. 8E, F). On the other hand, we detected lower levels of proton signals of ω-3 fatty acid docosahexaenoic acid and its derivatives, which lead to pro-resolving mediators (SPMs) [20] endowed with anti-inflammatory capabilities (Fig. 8E, F). The analysis of the polar fractions (Supplementary Fig. 6A-C) highlighted high levels of the proton signals of choline in SULT2B1b-LLC tumors, an observation strictly correlated with the low levels of the proton signals of phospholipids (Fig. 8B, C). Indeed, choline is an essential nutrient for all the cells and plays a role in the synthesis of the phospholipid components of cell membranes [41]. Furthermore, we observed higher levels of the proton signals of glycine in SULT2B1b-LLC tumors (Supplementary Fig. 6C) that could be correlated to the lower levels of the proton signals of cholesterol (Fig. 8B, C), as the glycine is linked to cholesterol transport in the bile acid metabolism pathway [42]. Similar results were also detected on the polar fractions of tumors treated with PFM037 (Supplementary Fig. 6D, E), indicating that SULT2B1b and PFM037 induce similar lipidomic and metabolomic changes in tumors.

### PFM037 synergizes with immunotherapy to enhance antitumor responses

**Fig. 9:**
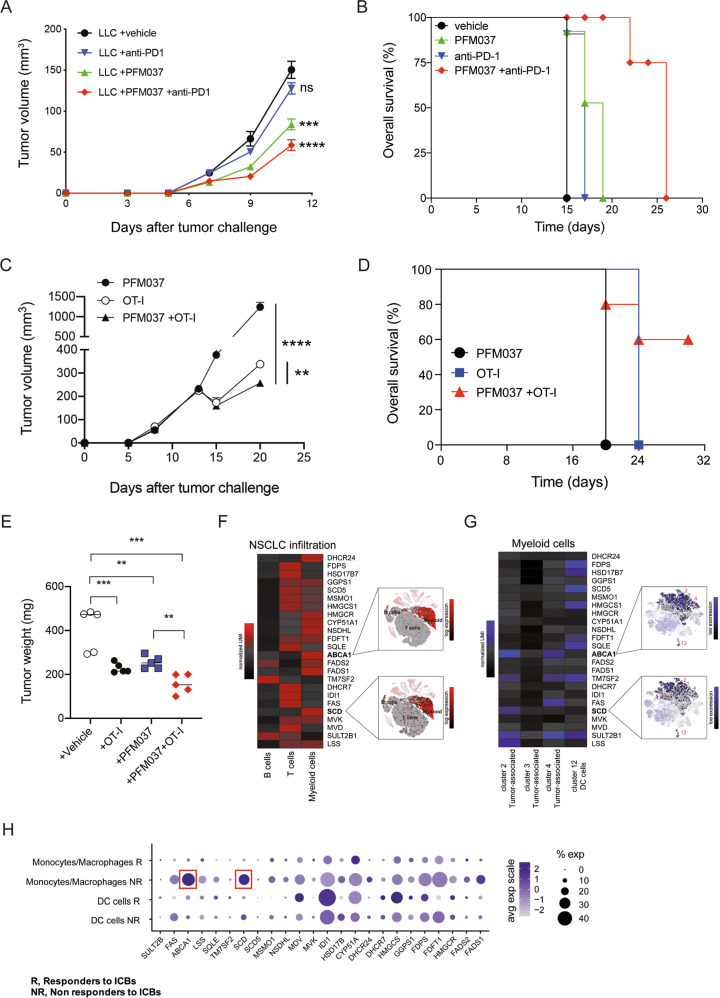
Synergizing effects of PFM037 and immunotherapy in controlling lung and melanoma tumor growth and analysis of myeloid cells infiltrating human tumors. **A** LLC tumor growth in mice treated with PFM037, anti-PD-1 mAb, the combination therapy, or vehicle plus ctrl mAb (*n* = 5 mice/group). ***P < 0.001; ****P < 0.0001; ns, not significant (Student’s t-test). **B** Overall survival of LLC-bearing mice treated with PFM037, anti-PD-1 mAb, the combination therapy, or vehicle plus ctrl mAb (*n* = 6 mice/group). Statistical comparison was performed by log-rank test (vehicle vs anti-PD1, *P* = 0.0051; vehicle vs *P*FM037, *P* = 0.0051; vehicle vs *P*FM037 + anti-PD1, *P* = 0.0009). **C, D** Tumor volume (**C**) and overall survival (**D**) of NOD-SCID mice challenged with LLC-OVA and treated with PFM037 and/or adoptive cell therapy with in vitro activated OT-I T cells (*n* = 5 mice/group). For tumor volume **P = 0.0018; ****P = 0.0001 (Student’s t-test). For overall survival statistical comparison was performed by log-rank test (PFM037 vs OT-I, *P* = 0.0027; PFM037 vs *P*FM037 + OT-I, *P* < 0.014; OT-I vs PFM037 + OT-I, not significant). **E** B16F1-OVA tumor weight following the treatment with vehicle, PFM037, in vitro activated OT-I alone, or in combination with PFM037 (*n* = 5 mice/group). **P < 0.01; ***P < 0.001 (Anova). **F** Average expression heatmap (normalized-UMI) of reverse cholesterol transport (RCT)/cholesterol synthesis genes in NSCLC immune-infiltrating cell populations [43]. Insert displays t-SNE plots of all cells profiled, color-coded according to the expression of *ABCA1* and *SCD* transcripts in the same populations. **G** Average expression heatmap (normalized-UMI) of LXR/cholesterol synthesis genes in tumor-associated monocyte/macrophage and DC subsets of myeloid cells [43]. Insert displays t-SNE plots of myeloid cells profiled, color-coded according to the expression of *ABCA1* and *SCD* transcripts in the same populations. (**H**) Dot plots of LXR/cholesterol synthesis genes in melanoma-infiltrating monocytes and DCs profiled, divided in Responder and Non-Responder patient-cells [44]. Each dot is colored according to the expression value and the size is related to the percentage of cells expressing the gene in the population of interest.

We reasoned that PFM037 by increasing the intratumor number of monocyte-derived APCs could synergize with immunotherapy. Therefore, we treated LLC-bearing mice with PFM037 and anti-PD-1 mAbs and observed a heightened control of tumor growth (Fig. 9A), and a prolonged overall survival with the combination therapy (Fig. 9B), suggesting that PFM037 could be used alone or in combination with immune checkpoint blockers (ICBs) (e*.*g. anti-PD-1 mAb) for cancer treatment. Moreover, we observed a better control of tumor growth (Fig. 9C) and a prolonged survival (Fig. 9D) of NOD-SCID mice bearing LLC-OVA tumors receiving the combination of PFM037 and the adoptive transfer of in vitro activated OT-I T cells. Finally, we observed a better control of tumor growth (Fig. 9E) in immunocompetent mice bearing the melanoma B16F1-OVA and treated with the combination of PFM037 and in vitro activated OT-I T cells. Thus, indicating that SULT2B1b/PFM037 treatments reprogram the hostile tumor microenvironment into an immune-sensitive one.

### Expression of LXR/cholesterol genes in myeloid cells infiltrating human tumor samples predicts tumor response to ICBs

To find molecular correlates in human tumors we evaluated the expression and clinical significance of LXR/cholesterol synthesis genes in myeloid cells infiltrating human tumors by taking advantage of recently published scRNA-seq datasets of non-small cell lung cancer (NSCLC) [43] and melanoma samples, the latter from ICB-responder and non-responder patients [44]. We detected an increased expression of *ABCA1* and *SCD* transcripts, as well as cholesterol synthesis genes, such as *HMGCR*, in myeloid cells infiltrating NSCLC samples (Fig. 9F). We observed the expression of *ABCA1* and *SCD* mainly in tumor-associated macrophages (cluster 2) (Fig. 8G), while *HMGCR* was mainly expressed in DCs (Fig. 9G). To correlate myeloid cell infiltration, LXR/cholesterol synthesis pathways and response to immunotherapy, we interrogated the scRNA-seq dataset of melanoma samples of ICB-responder and non-responder patients [44] and observed a statistically significant enrichment of *ABCA1* and *SCD* transcripts in monocytes/macrophages of patients not responding to ICBs (Fig. 9H and Supplementary Table 3) (*ABCA1* NR vs R = 1.52; *P* = 4.29 × 10^-8^; *SCD* NR vs R = 1.80; *P* = 2.93 × 10^-5^; Wilcoxon rank sum test). Of note, the enrichment of *ABCA1* transcripts in monocytes/macrophages of non-responders turned out to be the most significant among all melanoma-infiltrating CD45^+^ cells (T lymphocytes, B lymphocytes, etc.; *ABCA1* NR vs All = 1.40; *P* = 5.79 × 10^-212^; Wilcoxon rank sum test) (Supplementary Fig. 7 and Supplementary Table 4). We also observed the enrichment of some cholesterol synthesis genes, such as *HMGCS1*, in DCs of ICB responder patients, although it was not statistically significant (Fig. 9H and Supplementary Table 5). These results indicate that tumor-infiltrating myeloid cells (monocytes and DCs) differentially express genes associated to LXR and cholesterol synthesis pathways and that the over-expression of transcripts associated to LXR activation in monocytes/macrophages correlates with a worse clinical outcome to ICBs.

Altogether, these results indicate that SULT2B1b and PFM037 perturbation reprogram the tumor microenvironment generating an immune contexture, i.e. monocyte-derived APCs (Supplementary Fig. 8), contributing to antitumor immune responses and synergizing with immune checkpoint inhibitors as well as adoptive cell therapy.

## Discussion

Histopathologic pictures characterized by low intratumor numbers of T cells and dendritic cells (DCs) frequently correlate with the failure of immune checkpoint blockers (ICBs) therapy in advanced cancer patients [2]. This histopathologic condition often associates with high intratumor number of immature myeloid cells, as well as high levels of VEGF, M-CSF and IL-6, which are known to block the differentiation of cDC [4, 45]. Although the paralysis of the antigen presentation due to the blockade of cDC differentiation argues against the occurrence of antitumor immune responses in advanced cancer patients, the identification of *de novo* generated TCR clonotypes recognizing cross-presented antigens following ICB therapy [46], predicts the residual activity of rare DCs not yet dysfunctional, or the presence of surrogate antigen-presenting cells. Although recent studies have pointed out the key role played by resident cDCs, particularly cDC1, to inducing both CD8^+^ and CD4^+^ TAA-specific T cells [36, 47], there are other studies demonstrating that monocyte-DCs can be a relevant source of antigen-presenting cells (APCs), capable of priming antigen-specific T cells in vivo [18, 19, 25, 48]. Different mechanisms/factors, such as chemotherapeutic agents [18], transcription factors [19], retinoids [48], have been shown to favor the differentiation of monocyte-DCs. Here, we demonstrate that also the perturbation of the reverse cholesterol transport pathway (i.e., the LXR/oxysterol pathway), enhances intratumor monocyte-derived APC activation and differentiation (Supplementary Fig. 8), which independently of their capacity to ultimately interact with antigen-specific T cells, induce effective antitumor immune responses, as shown by our experiments performed under CCR2 blockade. Circulating monocytes have been widely used in the past to obtain large numbers of human monocyte-DCs for the immunotherapy of advanced cancer patients [49]. These cells, loaded with TAA and injected into patients induced suitable TAA-specific immune responses, which in some cases resulted in optimal antitumor responses [49–51]. We observed monocyte-DCs phagocytosing tumor-derived mCherry^+^ bodies in tumor-draining lymph nodes (Fig. 4F); however, it remains to evaluate whether these cells prime efficiently tumor-specific T cells either directly or indirectly.

ICBs are standard of care for several tumor types (melanoma, NSCLC, head and neck cancers, etc.) [1], and adoptive cell therapy is rapidly emerging as a powerful immunotherapy strategy [52]. In our pre-clinical models, the concomitant administration of PFM037 and immunotherapy with either anti-PD-1 mAb or adoptive cell therapy enhanced the antitumor responses. Indeed, in both conditions we observed a prolonged survival of tumor-bearing mice treated with the combo therapies, indicating that the perturbation of the reverse cholesterol transport process improves the effects of immunotherapy by overcoming mechanisms of immune escape, such as the low intratumor number of APCs. These results are in accordance with bioinformatic analyses showing improved antitumor responses in melanoma patients with lower levels of LXR target gene transcripts*,* i.e*., ABCA1* and *SCD* (Fig. 9H). Therefore, our findings highlight the possibility to overcome resistance mechanisms related to the low number of tumor-infiltrating APCs and to augment the clinical activity of immunotherapy by manipulating LXR/SULT2B1b axis.

The results of the GSEA showing a differential expression of metabolic pathways belonging to cholesterol and fatty acid metabolisms in monocyte-DCs and Ly6C^low^ cells isolated from LLC-SULT2B1b tumors, together with both the analysis of lipid levels in the blood of mice treated with PFM037 (Fig. 5N) and previous results demonstrating that SULT2B1b interferes with LXR and lipid metabolism [11], enabled us to evaluate the lipidomic profiles of tumors perturbed by SULT2B1b or PFM037. PFM037 is a novel LXR antagonist, structurally and functionally analog to endogenous sulfate oxysterols. The comparative analysis of the effects exerted by SULT2B1b and PFM037 in the tumor microenvironment highlighted some commonalities, i.e., the reprogramming of cholesterol/cholesterol products, phosphatidylcholine and phosphatidylethanolamine, as well as fatty acids endowed with inflammatory (arachidonic acid) or anti-inflammatory (docosahexaenoic acid) activities. Since arachidonic acid is the precursor of lipid mediators of the acute phase of inflammation (i*.*e., prostaglandins, leukotrienes, eoxins) while docosahexaenoic acid is an ω-3 FA with anti-inflammatory properties [20], our findings suggest that tilting the balance towards lipids mostly involved in the acute phase of inflammation may contribute to monocyte-derived cell activation and differentiation, including monocyte-DC differentiation. It remains to establish the type(s) of lipid mediators contributing to monocyte-derived cell activation and differentiation, given the heterogenous nature of lipids and the multitude of effects they exert depending on their nature [53].

We show that the in vitro and in vivo effects induced by PFM037 are mainly dependent on LXR signaling, as indicated by in vitro differentiation experiments of *Lxrαβ*^*-/-*^ monocytes in the presence or in the absence of PFM037, and by tumor challenge experiments in *Lxrαβ*^*-/-*^ bone marrow chimera mice treated with vehicle or PFM037. However, in both experiments we noticed phenotypic and functional features predicting some LXR-independent effects by PFM037. In vitro, we observed an increased number of *Lxrαβ*^*-/-*^ monocytes acquiring CD11c molecules when LXR-deficient monocytes were treated with PFM037 (Fig. 7D). In vivo, we observed an earlier control of tumor growth when wild-type and *Lxrαβ*^*-/-*^ chimera mice were treated with PFM037 (Fig. 7C). Further studies are needed to clearly investigate the role of LXR-dependent and -independent effects of PFM037 treatment.

There is still debate on the role played by LXR on tumor growth, i.e. protumor vs antitumor effects. Molecular and cellular clues, such as the presence of two isoforms of the receptor, LXR-α and -β, which can be selectively expressed by tumoral and non-tumoral cell components of the tumor microenvironment, and the sensitivity of different tumor-infiltrating immune cells to LXR activation/repression, might therefore explain some contrasting results obtained by different groups. Our work is for instance in accordance with studies showing that the pharmacologic inhibition of LXR by the inverse agonist SR9243, enhances dendritic cell activity that is suppressed by tumor-produced LXR ligands, and induces in vivo control of triple-negative breast cancers (TNBC) through the stimulation of CD8^+^ T-cell cytotoxic activity [54, 55]. Interestingly, Flaveny and colleagues reported upregulated levels of the LXR target gene *ABCA1* in myeloid cells (macrophages and dendritic cells) infiltrating human TNBC samples [55], an observation in agreement with our results in human lung and melanoma samples (Fig. 9F-H). While these studies point to the detrimental effects of LXR activation, other studies have instead shown effective antitumor responses when LXRs are activated in vivo by the administration of LXR agonists [56, 57]. These studies have reported the downregulation of the frequency of intratumor myeloid-derived suppressor cells (MDSCs) and increased CTL activity in response to high doses of LXR agonists [56], and the reduction of intratumoral regulatory T cells (Treg) depending on the downregulation of the Treg-attracting chemokine *Ccl17* by MHC-II^high^ tumor-associated macrophages in response to lower doses of LXR agonists [57]. In our experimental settings, we sought to fix some variables by transplanting tumors in *Lxrαβ*^*-/-*^ bone marrow chimera mice to rule out the possible interference of *Lxrαβ*^*-/-*^ non-hematopoietic stromal cells. Further studies are however needed to dissect in a systematic manner the complex role of LXRs in the tumor microenvironment, i.e. generating or taking advantage of LXR-deleted cell/tissue-specific mice and by using LXR antagonists, such as PFM037, and selective LXR agonists [58].

SULT2B1b perturbation induces SREBP2 activity [12] and therefore the intracellular accumulation of cholesterol in APCs. This pathway, by promoting the inflammasome-dependent IL-1β release [59], has been shown to lead to APC activation [60]. Although at early times we did not observe difference in IL-1β release after PFM037 treatment (*Russo et al. unpublished observations*), we cannot rule out the possibility that it may occur at later times, further contributing to monocyte-derived cell activation and differentiation and to the generation of robust antitumor immune responses.

In the microenvironment of SULT2B1b-tumors or PFM037-treated tumors we detected higher numbers of monocyte-DCs and a heterogenous population of monocyte-derived cells resembling inflammatory monocytes or activated macrophages, which expressed higher levels of transcripts associated to the antigen presentation (*H2-Ab, H2-Eb, H2-Aa, H2-DMa, H2DMb, Cd74 and Ciita*) and to T-cell recruitment (*Cxcl9* and *Cxcl10*) [26]. Although these cells when pulsed with the SIINFEKL peptide, were much less stimulators than pulsed monocyte-DCs (Supplementary Fig. 2C-E), we cannot exclude that they contribute to the antitumor immune response by recruiting antitumor T cells and/or generating a stimulatory immune contexture.

In conclusion, our findings indicate that SULT2B1b-derived products reprogram the lipidome of the tumor microenvironment and favor monocyte-derived cell activation and differentiation, including monocyte-DCs (Supplementary Fig. 8), by interfering with LXR signaling. The characterization of a new compound, obtained by the chemical sulfation of the LXR-inactive oxysterol 22S-HC and promoting effective antitumor responses, may represent an important step towards the clinical translation of new compounds linking lipid metabolism and immunity, given alone or in combination with immunotherapy.

## Materials and methods

### Animal studies and reagents

C57BL/6 CD45.1, CD45.2, transgenic OT-I and OT-II mice were from Charles River (Calco, Italy). *Ccr2*^*-/-*^ mice were from Jackson Laboratory. Mice were maintained in the pathogen-free facility of San Raffaele Scientific Institute. Experiments were conducted in compliance with the Institutional Animal Care and Use Committee programme (IACUC n° 656, 657 and 835) and in accordance with ARRIVE guidelines. Lewis Lung Carcinoma (LLC) cell line, LLC-Mock, LLC-SULT2B1b, LLC-OVA and LLC-mCherry were maintained in DMEM complete medium (10% FBS supplemented with penicillin, streptomycin, glutamine and Hepes). Tumor cell lines were from ATCC and were tested for mycoplasma contamination. RMA was in RPMI complete medium and B16F1-OVA in DMEM complete medium. 25-Hydroxycholesterol-3-sulfate (25-HC-3S) was from Avanti Polar Lipids. Carboxyfluorescein succinimidyl ester (CFSE) was used at 5 μM (Molecular Probes). MHC-I-restricted SIINFEKL (257-264) and MHC-II-restricted ISQAVHAAHAEINEAGR (323-339) OVA peptides were from InvivoGen. Monocyte Isolation Kit (Miltenyi). Click-it EdU flow cytometry assay kit (Thermofisher).

### PFM037 synthesis

**Scheme 1:**
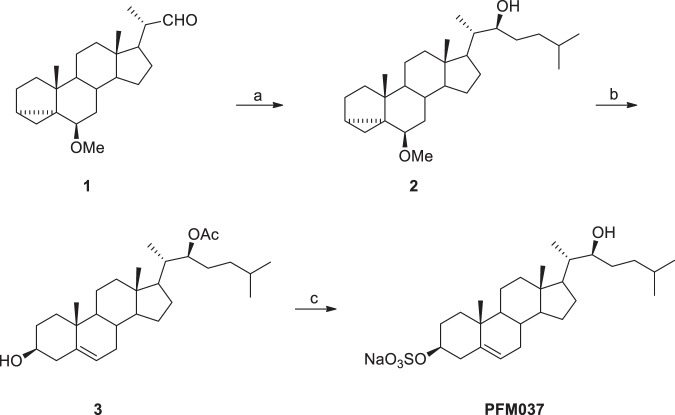
Synthesis of 22(*S*)-Hydroxycholesterol-3-sulfate sodium salt (PFM037)^a^. ^a^Reagents and conditions: (a) isoamyl bromide, Mg, 0 °C to rt, 5 h; (b) i. Ac_2_O, 4-DMAP, CH_2_Cl_2_, pyridine, rt, 20 h; ii. *p*TsOH, dioxane/H_2_O, 80 °C, 1 h; (c) i. SO_3_-pyridine complex, pyridine, rt, 4 h; ii. 1.25 M NaOH in MeOH, reflux for 1 h, then rt overnight.

PFM037 (22(*S*)-hydroxycholesterol-3-sulfate sodium salt) was synthesized starting from 6β-methoxy-3α,5-cyclo-23,24-dinor-5α-cholan-22-al (**1**) [61] in four steps and 26.8% overall yield Scheme 1.

#### 22(S)-6β-Methoxy-3α,5-cyclo-5α-cholest-22-ol (2)

A solution of isoamyl bromide (0.65 g, 4.31 mmol) in THF (1.5 mL) was added dropwise to a stirred suspension of magnesium turnings (0.11 g, 4.83 mmol) in THF (3 mL) and the resulting mixture was kept at 0 °C under argon atmosphere. After 1 h, a solution of **1** (0.50 g, 1.45 mmol) in THF (3 mL) was added dropwise and the mixture was allowed to reach rt. After 4 h, a saturated solution of NH_4_Cl (15 mL) was added and the mixture extracted with EtOAc (2 ×15 mL). The combined organic layers were washed with brine (10 mL) and dried over anhydrous Na_2_SO_4_. After the removal of the solvent, the crude product, thus obtained, was purified by flash chromatography (light petroleum/EtOAc, 95:5) affording **2** in 66% yield. ^1^H NMR (400 MHz, CDCl_3_) δ 0.43 (s, 1H), 0.65 (s, 1H), 0.73 (s, 3H), 1.87-1.99 (m, 3H), 2.77 (s, 1H), 3.32 (s, 3H), 3.63 (s, 1H). ^13^C NMR (100 MHz, CDCl_3_) δ 11.43, 12.17, 13.04, 19.26, 21.43, 22.54, 22.69, 22.76, 24.09, 24.92, 27.79, 28.17, 30.52, 33.19, 33.30, 35.03, 35.18, 35.63, 40.12, 40.24, 42.66, 43.33, 47.93, 52.67, 56.36, 56.54, 73.88, 82.35.

#### 22(S)-Acetoxycholest-5-en-3β-ol (3)

Acetic anhydride (0.08 g, 0.83 mmol) and 4-DMAP (0.01 g, 0.10 mmol) were added to a stirred solution of **2** (0.16 g, 0.40 mmol) in CH_2_Cl_2_ (15 mL) and pyridine (0.06 mL) and the resulting mixture was kept at room temperature under argon atmosphere. After 20 h, a solution of 3 M HCl (10 mL) was added and the organic layer was separated and washed with H2O (15 mL), brine (15 mL) and then dried over anhydrous Na2SO4. After the removal of the solvent the crude product was dissolved in dioxane/water (3:1, 8 mL) and *p*-toluenesulfonic acid (0.01 g, 0.04 mmol) was added to the resulting stirred solution. After heating at 85 °C for 1 h, the mixture was concentrated in vacuo, water (5 mL) was added and the mixture extracted with EtOAc (2 × 7 mL). The combined organic layers were washed with brine (10 mL) and dried over anhydrous Na_2_SO_4_. After the removal of the solvent the crude product was purified by flash chromatography. Elution with light petroleum/EtOAc, (80:20) gave **3** in 70% yield. ^1^H NMR (400 MHz, CDCl_3_) δ 0.59 (s, 3H), 0.77 (s, 3H), 0.79 (s, 3H), 0.91 (s, 3H), 1.95 (s, 3H), 2.10-2.25 (m, 2H), 3.44 (m, 1H), 4.84 (t, 1H, *J* = 6.7 Hz), 5.26 (bs, 1H); ^13^C NMR (100 MHz, CDCl_3_) δ 11.62, 12.69, 19.36, 21.04, 21.25, 22.40, 22.66, 24.20, 28.02, 28.09, 29.96, 31.57, 31.78, 31.87, 34.89, 36.43, 37.20, 38.89, 39.67, 42.21, 50.03, 52.47, 56.57, 71.71, 76.56, 121.65, 140.65, 171.00.

#### 22(S)-Hydroxycholesterol-3-sulfate sodium salt (PFM037)

Sulfur trioxide-pyridine complex (0.12 g, 0.76 mmol) was added to a stirred solution of **3** (0.16 g, 0.38 mmol) in pyridine (1.5 mL) and the resulting mixture was stirred at rt under argon atmosphere. After 4 h cold light petroleum (2 mL) was added and the precipitate, thus formed, was separated by vacuum filtration and then washed with cold light petroleum. The solid was then suspended in 1.25 M NaOH in MeOH (3 mL) and the resulting mixture heated at reflux for 1 h. After further 12 h of stirring at rt, H_2_O (0.5 mL) was added and the resulting precipitate isolated by vacuum filtration, washed with H_2_O and acetone and finally dried to afford PFM037, as a white solid, in 58% yield: mp = 182.2–183.3 °C; ^1^H NMR (400 MHz, DMSO-d_6_) δ 0.56 (s, 3H), 3.31 (s, 1H), 3.76 (s, 1H), 5.20 (s, 1H); ^13^C NMR (100 MHz, DMSO-d6) δ 11.96, 12.07, 19.37, 20.94, 22.87, 23.00, 27.61, 28.00, 29.08, 31.82, 33.19, 35.81, 36.39, 37.17, 41.94, 49.87, 52.25, 56.58, 71.67, 75.68, 121.47, 140.96.

#### Immunofluorescence staining and confocal analysis

Tissues were harvested and fixed for 10 minutes in 4% (w/v) PFA (Sigma-Aldrich), then washed in PBS and dehydrated overnight in 30% sucrose (Sigma-Aldrich) at 4 °C. Samples were embedded in Tissue-Tek OCT compound (Bio-optica) and frozen in ethanol dry-ice bath (using Dehyol 95; Bio-optica). 7–10 μm thick sections were placed onto glass slides (Bio-optica), fixed in cold acetone for 5 minutes, dried, and kept at −80 °C until used. Slides were incubated 30 min with a blocking solution of PBS at 0.5% FBS and 0.05% Tween 20 (VWR) (PBS-T 0.05%), followed by primary specific antibodies or secondary reagents. Immunofluorescence images were acquired using Zeiss Axio Observer.Z1 microscope. Confocal images were acquired using Leica TCS SP8 microscope. Digital images were recorded in separately scanned channels with no overlap in the detection of emissions from the respective fluorochromes. Final image processing was performed with Adobe Illustrator CS6 and Adobe Photoshop CS6. The following antibodies and detection reagents were used: CD11b-APC (M1/70 clone) and CD3-PE (17A2 clone) and CD45.1-FITC (A20 clone) from Biolegend; mCherry (NBP2-25158 Novusbio); biotinylated CD11c (HL3 clone, hamster) from BD Bioscience and Streptavidin 488 or 647 (Thermo Fisher, Invitrogen). For quantification analysis, a total of 64 pictures taken on 5 LLC-Mock-bearing mice and 79 pictures taken on 5 LLC-SULT2B1b-bearing mice were analyzed for T-cell numbers and T-cell-DC clusters. Draining lymph nodes were harvested, fixed and processed as described above.

#### Macroscopic liver tumor quantification and Immunohistochemistry

At time of autopsy, visible macroscopic tumors were counted, and each liver macroscopically photographed. Livers were then perfused with PBS, harvested and different pieces were sampled, fixed in zinc-formalin and embedded in paraffin for hematoxylin/eosin or immunohistochemical analysis as described [62]. Immunohistochemical staining was performed using a Bond RX Automated Immunohistochemistry system (Leica Microsystems GmbH, Wetzlar, Germany) on 3-μm-thick sections. First, tissues were deparaffinized and pre-treated with the Epitope Retrieval Solution [ER1 Citrate Buffer for GFP (dilution 1:500, GFP Polyclonal Antibody, Thermo Fisher Scientific, Cat# A11122, RRID:AB_221569). After washing steps, peroxidase blocking was carried out for 10 min using the Bond Polymer Refine Detection Kit DS9800 (Leica Microsystems GmbH). Then, tissues were washed and incubated for 1 h RT with the primary antibody diluted in IHC Antibody Diluent (Novocastra, Leica RE7133). Subsequently, tissues were incubated with polymer-HRP (Biocare Medical, RT517H), developed with DAB-Chromogen for 10 minutes and counterstained with Hematoxilin for 5 minutes. For image acquisition and analysis eSlide Manager (Aperio Leica Biosystems) was used. All images were acquired using the Aperio AT2 system (Leica Biosystems). A total area of 97,6 mm^2^ (Vehicle) and 104,98 mm^2^ (PFM037) of liver was analyzed for each group of mice. Tumor cell quantification (MC38-GFP) was performed by automated image analysis software through the Color Deconvolution Algorithm v9.1 of the ImageScope program (version 12.4.3), following manufacturer’s instructions (Leica Biosystems). The images shown were identified as representative area of interest within the total area of the specimen and exported as ImageScope snapshots at 20x magnifications as described [38].

#### Analysis of tumor-infiltrating cells by flow cytometry

Mice were challenged with LLC, LLC-Mock, LLC-SULT2B1b and RMA tumor cells (*see* *tumor challenge experiments for details*). Tumors collected 7, 10, 12 or 14 days after injection were cut into small fragments and digested with Liberase TL (66 μg/ml, Roche) and DNAse I (150 μg/ml, Roche) for 25-30 minutes at 37 °C. Digested tissues were filtered by using a 75 μm diameter cell strainer. Single-cell suspensions were stained with Live/Dead Violet kit reagents (Molecular Probes) for 30 minutes at 4 °C. After washing, the cells were incubated for 5–10 minutes at RT with Fc-blocking solution (Anti-CD16/32, 10 μg/ml mouse Fc Block, BD) and then stained with various antibodies incubated at 4 °C for 20 minutes. The following antibodies were used: mAbs against mouse CD45 (30F11), CD45.1 (A20), CD45.2 (104), CD11b (MI/70), Ly6C (HK1.4), Ly6G (1A8), CD11c (N418), CCR2, MHC-II (M5/114.152), CD43 (S11), CD3 (145-2c11), CD8 (53-6.7), IFNγ (XMG1.2), CD19 (6D5), CD49b (DX5) and Ter119, CD26, CD64, MAR-1 all from Biolegends. CD209a (5H10), CCR7 (4b12) and F4/80 (BM8) were from eBioscience. Rat IgG, Rat IgG2a, Rat IgG2b, Rat IgM and Hamster isotype control mAbs were used as controls (Biolegends and BD Biosciences). For intracellular staining, cells were incubated with PMA (100 ng/ml) and ionomycin (500 ng/ml) in the presence of 10 μg/ml of Brefeldin for 4 h. Cells were then fixed using the IC Fixation buffer (eBioscience), permeabilized with 1x Permeabilization buffer (eBiosciences) and stained with anti-IFNγ mAb. Samples were run by FACSCanto flow cytometer and analyzed by FlowJo software gating on live cells. FACS sorting was performed by using the FACSAria Fusion (BD).

#### RNA-seq experiments

FACS-sorted monocyte-DCs and Ly6C^low^ cells were collected in complete RPMI and pelleted. Total RNA was extracted using RNA spin columns (PureLink RNA Mini Kit, Ambion). RNA quantification was carried out using Qubit Fluorimetric Quantification (Life Technologies) and 500 ng of RNA were used to prepare the library. Sequencing libraries were prepared following SMART-Seq v4 Ultra Low Input RNA (TaKaRa) protocol and Illumina TRUSEQ stranded protocol. Briefly, 1 ng of total RNA was used for cDNA synthesis, followed by PCR amplification. Quality and quantity of cDNA was checked running samples in DNA HS chip (Bioanalyzer 2100, Agilent). 500 pg of cDNA from each sample were used for tagmentation, followed by PCR and barcoding (Nextera XT, Illumina). Sequencing was performed using an Illumina Nextseq 500 with a High Output flow cell, 75 nt read length, to obtain an average of 30 M reads for each sample. Sequencing quality control and read alignment were obtained using the FastQC software to assess the quality of the fastq files [Andrews S. (2010). FastQC: a quality control tool for high throughput sequence data. Available online at: http://www.bioinformatics.babraham.ac.uk/projects/fastqc]. Reads were aligned to the mouse genome, version mm10, using STAR_2.5.3 [63] and the quality of the alignment assessed with bamtoools [64]. The Rsubread package [65] was used to assign read counts to the genes of the GENCODE basic gene model, version M13 (https://www.gencodegenes.org), with all isoforms of each gene considered together.

#### Differential expression of RNA-seq datasets

Genes with at least 1 cpm (count per million) in at least the number of samples of the group having the minimum (e*.*g. 3 samples if comparing a group of 3 samples vs a group of 3 or 4 samples) were considered expressed and assessed for differential expression. Differentially expressed genes were identified using limma [66]. Pairwise comparisons between the experimental groups (e*.*g. LLC-SULT2B1b and LLC-Mock conditions) were performed independently for monocyte-DCs and Ly6C^low^ cells. Genes with nominal *P* value < 0.01 and absolute value of log2 fold change > 1 were considered differentially expressed [67].

#### Gene set enrichment analysis (GSEA)

GSEA [68] (http://www.broad.mit.edu/gsea/) was used to analyze the expression profile of a gene signature associated to cholesterol metabolism as reported in [32].

#### Comparing transcriptomes of monocyte-DCs and Ly6C^low^ cells with public datasets

Gene expression profiles derived from RNA-Seq experiment were integrated with 4 datasets present in GEO (Gene Expression Omnibus) Database: GSE149619 [24], GSE90471 [23], GSE74427 [25] and GSE49358 [29]. Probeset-level data obtained from GEO were summarized at the gene level, then the gene expression profiles of 10,080 genes evaluated in both the public datasets and expressed in our RNA-seq experiments was considered for the integrative analysis using R and Bioconductor packages (https://www.R-project.org/) [69]. Data have been quantile normalized using limma [66] and samples clustered using hierarchical clustering algorithm with Spearman correlation-based distance in R Principal Component Analysis (PCA) has been performed on scaled data using [70]. Uniform Manifold Approximation and Projection (UMAP) has been done on the integrated dataset analyzed with Seurat [71]. Ten PCA components have been used in graph-based clustering, evaluated at different resolutions to identify stable clusters, then fixed at the value of 1 and represented in a bidimensional UMAP plot.

#### Extraction of the lipidic and polar fractions from tumor tissues

Frozen tissue of LLC-Mock, LLC-SULT2B1b and LLC + PFM037 tumors (*n* = 5 tumors/group) were cut to an appropriate size (mean weight: 10 mg) and placed in microcentrifuge tubes. They were re-suspended in 170 µL of H_2_O and 700 µL of methanol and sonicated for 60 sec. Then, we added 350 µL of chloroform and mixed the samples on an orbital shaker in ice for 10 minutes. After that, 350 μL of H_2_O/chloroform (1:1, v/v) were added to each suspension and centrifuged at 4000 rpm for 10 minutes at 4 °C. Thereafter, the aqueous (polar) and lipidic (non-polar) phases were collected separately, transferred to a glass vial and evaporated.

#### ^1^H NMR spectra acquisition and data analysis

All the lipidic and polar samples were analyzed using Nuclear Magnetic Resonance (NMR). The lipidic fractions were dissolved in 700 µL of deuterated chloroform (CDCl_3_) and 0.03 volume percentage of tetramethylsilane (TMS) and inserted in NMR tubes whereas the polar fractions were dissolved in 630 µL of PBS-D_2_O with the pH adjusted to 7.20, and 70 µL of trimethylsilylpropanoic acid (TSP) (1% in D_2_O). TMS and TSP were used as the internal standards. A 600-MHz Bruker Avance spectrometer with a TCI cryoprobe was used to acquire ^1^H spectra on all the lipidic and polar fractions at 300 K for 512 transients using the excitation sculpting pulse sequence to suppress water resonance in the case of polar fractions. All ^1^H NMR spectra were manually phased and baseline-corrected and referenced to the CH_3_ resonance of TSP or TMS at 0 ppm. The spectral 0.50-8.60 ppm region of ^1^H-NMR spectra was integrated in buckets of 0.04 ppm by AMIX package (Bruker, Biospin, Germany). In details, we excluded, in the case of the polar spectra, the water resonance region (4.5–5.2 ppm) during the analysis and normalized the bucketed region to the total spectrum area using Pareto scaling by MetaboAnalyst v4.0 tool [72]. The partial least squares-discriminant analysis (PLS-DA) algorithm was applied to explain the maximum separation between the defined class samples in the data. PLS-DA is a multivariate regression method that extracts linear relationships from two data block, X (NMR bucket variables) and Y (information data), by reducing the structured noise and highlighting the maximum correlation between the two matrices. Score, Loading and Variable Importance in Projection (VIP) Plots were used to highlight and assess the role of X-variables (NMR signals) in the classification models and, hence, to prioritize the discriminating peaks for identification. We show in Loading and VIP plots the top 10 more different NMR signals obtained comparing the different groups. Metabolite assignments were based on the comparison of chemical shifts and spin-spin couplings with reference spectra present in the human metabolome database (HMDB) [73]. The normalized proton signals of the statistically significant metabolites were reported by box-plot graphs.

#### Tumor challenge experiments

C57BL/6 mice were injected subcutaneously with LLC-Mock (1 × 10^6^), LLC-SULT2B1b (1 × 10^6^), RMA (2 × 10^5^), or B16F1-OVA (2 × 10^5^) tumor cells. Mock- and SULT2B1b-transduced tumor cells have been described in [7, 74]. We evaluated tumor size measuring perpendicular diameters by a caliper every 2–3 days. Data are reported as the average tumor volume ± SD. In selected experiments tumors were injected in *Ccr2*^*-/-*^ mice. Tumor challenge experiments in *Lxrαβ*^*-/-*^ bone marrow chimera mice. To establish bone marrow chimera mice, we transplanted lethally irradiated (11 Gy) C57BL/6 mice with the bone marrow of *Lxrαβ*^*-/-*^ or WT mice (5 × 10^6^ bone marrow cells/mouse). Six-eight weeks later, we challenged chimera mice with LLC and evaluated tumor size measuring perpendicular diameters by a caliper. *Lxrαβ*^*-/-*^ genotype was carried out by PCR on blood cells before tumor challenge. During the reconstitution phase, transplanted mice were treated with enrofloxacin for 15 days (7.5 mg/150 μl of Baytril 5% solution in 300 ml of drinking water) following the indication of the veterinary of our spf facility.

#### Treatment of tumor-bearing mice with LXR antagonists alone or with immunotherapy

Tumor-bearing mice were treated with 25-HC-3S or PFM037 6-7 days after tumor challenge. After 6-7 days, mice were randomly assigned to either treatment or vehicle groups. Mice were injected with 300 μg of either compound given i.v. for 5 days/week. For liver metastasis studies, eight-to ten-week-old male C57BL/6 mice were injected through the superior mesenteric vein, with 7 × 10^4^ GFP-expressing MC38 cells (H-2b, C57BL/6-derived, MC38GFP) as previously described [37, 38]. For mesenteric vein injections, deep anesthesia was induced by isoflurane inhalation (5% induction and 2% for maintenance in 2 l/min oxygen). The indicated number of MC38-GFP cells was injected into the superior mesenteric vein using a 29 G needle and to prevent excessive bleeding, vein puncture was compressed with a sterile and absorbable hemostatic gauze (TABOTAMP®). The peritoneum and skin were sutured with silk 4.0 and 7 mm wound clips as described [38]. In selected experiments, we used anti-PD-1 mAb (clone RMP1-14, In vivo BioXcell) 250 μg/mouse every three days, alone or in combination with PFM037. B16F1-OVA-bearing mice were treated with 400 μg of PFM037 for 5 days/week. For controls of mAb experiments, mice were injected with 250 μg of rat IgG2a isotype control antibody. For adoptive transfer studies, NOD-SCID mice challenged with LLC-OVA were treated with PFM037 (300 μg i.p. 3 days/week) 6 days after tumor injection. Three days later, mice were randomly assigned to either treatment or vehicle groups and infused i.v. with OT-I T cells (2 × 10^6^ OT-I/mouse) activated and expanded in vitro for 3-4 days with IL2 (20 IU/ml), IL27 (20 ng/ml) and MHC-I-restricted SIINFEKL (257-264) OVA peptide (2 μg/ml) in RPMI 10% FBS + 50 μM β-ME. C57Bl/6 mice challenged with B16F1-OVA were treated with PFM037 (400 μg i.p. 3 days/week) 6 days after tumor injection. Three days after, mice were randomly assigned to either treatment or vehicle groups and infused i.v. with OT-I T cells (2×10^6^ OT-I/mouse) activated in vitro for 7-8 days with IL2 (20 IU/ml) and MHC-I-restricted SIINFEKL (257-264) OVA peptide (2 μg/ml) in RPMI 10% FBS + 50 μM β-ME. Tumors were collected and measured 14 days after tumor challenge. Different from LLC and RMA tumor models, we used 400 μg of PFM037 to treat B16F1-OVA-bearing mice, as 400 μg of PFM037 induced an antitumor activity much stronger than 300 μg of PFM037. For the liver metastasis model, given the complexity of the model we started directly with 400 μg of PFM037 to minimize the number of mice to use.

#### Blocking experiments with anti-CCR2 mAb

Mice bearing 7 day-LLC-SULT2B1b tumors were treated every day i.p with 20 μg anti-CCR2 antibody [34] or rat IgG control antibody. Mice were sacrificed 12 days after tumor injection and analyzed for tumor weight and for the content of tumor-infiltrating monocyte-DCs

#### Single-cell RNAseq data analysis

Two published datasets were considered in this study: E-MTAB-6149 and E-MTAB-6653 for lung tumor microenvironment and GSE120575 for CD45^+^ infiltration in melanoma. For lung dataset, tSNE projections were obtained uploading loom files in SCope focusing first on all cell types profiled and then only on myeloid clusters. Moreover, the expression of the cholesterol synthesis signature genes (*FADS1, FADS2, HMGCR, FDFT1, FDPS, GGPS1, HMGCS1, DHCR7, DHCR24, CYP51A1, HSD17B7, IDI1, MVK, MVD, NSDHL, MSMO1, SCD5, SCD, TM7SF2, SQLE, LSS, ABCA1, FAS, SULT2B1*) was investigated at the population level using normalized-UMI counts only for tumor samples, focusing first on immune-infiltrating population (B cells, T cells and myeloid cells) and then on tumor-associated clusters of myeloid cell type (clusters 2, 3, 4 and 12). The melanoma dataset was processed retrieving normalized gene expression data, cluster and patient information from GEO database and Supplementary Material of the original paper. Then the expression matrix was imported and processed in Seurat package only for data visualization. Cluster and patient information (response to treatment with immune checkpoint blockers) was added to the dataset using “AddMetaData” function. Also in this case, the dataset was investigated for the expression of the cholesterol synthesis signature genes considering both the percentage and level of expression of the cells belonging to a specific cluster. Wilcoxon rank sum test has been adopted with FindMarkers function (logfc.threshold = 0.5, min.pct = 0.2, only.pos = TRUE) to verify if some cholesterol synthesis signature genes could be differentially expressed between different clusters of interest.

#### Cell lineage tracing experiments and in vivo cell proliferation assays

Monocytes were purified from the bone marrow of C57Bl/6 Ly.1 (CD45.1) mice using the Monocyte Isolation Kit (Miltenyi). The enriched population contained >95% of Ly6C^high^ monocytes. 1.5-2.0 × 10^6^ purified Ly6C^high^ monocytes were infused i.v. into *Ccr2*^*-/-*^ CD45.2 mice bearing 3-day established LLC-Mock, LLC-SULT2B1b or LLC-mCherry tumors. Twenty-four and 72 hours later, mice were sacrificed, and tumors evaluated for weight and for the content of monocyte-DCs. For cell proliferation experiments, *ccr2*^*-/-*^ mice (CD45.2^+^) were injected with 5 × 10^6^ tumor cells. After 3 days, mice received CD45.1^+^ monocytes. Twenty-four hours later, mice were injected i.p. with 1 mg/mouse of EdU. Four hours later, mice were sacrificed, tumor collected and digested as reported above. Cells were then stained, run by FACScanto (BD) and analyzed with FlowJo software. For the analysis of draining lymph nodes, *ccr2*^*-/-*^ mice (CD45.2^+^) were injected with 5 × 10^6^ tumor cells. After 3 days, mice received CD45.1^+^ monocytes and 48 hours later lymph nodes were collected and treated as reported in *immunofluorescence staining and confocal analysis* section.

#### Antigen presentation assays

Splenic OT-I or OT-II were purified with CD8a and CD4 (L3T4) microbeads (Miltenyi), respectively. Enriched OT-I or OT-II were labeled with CFSE (5 μM) and plated at a density of 1 × 10^6^/ml. Monocyte-DCs and Ly6C^low^ cells were FACS-purified from 10-day established LLC-SULT2B1b tumors and loaded with 10μg/ml of OVA-specific MHC-I and -II peptides to stimulate OT-I and OT-II T cells, respectively. Then, 1 × 10^4^ monocyte-DCs or Ly6C^low^ cells were co-cultured with 1 × 10^5^ OT-I or OT-II in RPMI complete medium. Cells were cultured at 37 °C for 3 days and analyzed by flow cytometry for CFSE dilution. Supernatants collected 3 days later, were tested by ELISA to measure the content of mouse IFN-γ in agreement with manufacturer’s instructions. T cell proliferation was expressed as proliferation index and CFSE-diluted cells. Proliferation index was analyzed and calculated by FlowJo software. OT-I and OT-II CFSE-diluted cells were presented as the percentage of OT-I and OT-II cells undergoing multiple rounds of proliferation. The percentage of CFSE-diluted OT-I and OT-II cells following activation with peptide-loaded Ly6C^low^ cells is relative to the percentage of CFSE-diluted OT-I and OT-II cells activated with peptide-loaded monocyte-DCs (set as 100%).

#### Reporter assay for nuclear receptor transcriptional activity

Human embryonic Kidney 293 cells (American Type Culture Collection) were cultured in Dulbecco’s Modified Eagle’s medium containing 10% of fetal bovine serum at 37 °C in humidified atmosphere of 5% CO_2_. We transiently transfected HEK293 cells (4 × 10^4^ cells per well) in 48 well plate with the reporter plasmids pMH100X4-TK-luc (100 ng/300μl), Renilla (22 ng/300μl) together with 100 ng/300μl of pCMX-Gal4-LXRα or pCMX-Gal4-LXRβ plasmids using X-tremeGENE 9 DNA Transfection Reagent (Roche). Six hours after transfection, we treated the cells with the appropriate compound for 24 hours. We analyzed luciferase activities by luciferase Dual Reporter Assay Systems (Promega) according to the manufacturer’s protocol. GAL4-LXRs, and TK-MHC100-luc plasmids were described in [7]. For mammalian two-hybrid assays the human liver cell line Hhu7 (American Type Culture Collection) was transiently transfected with the reporter plasmids p5xUAS-tk-Luc (100 ng/300μl) together with pGAL4-RAP250-Del4 (133 ng/300μl), described in [39], and either of pVP16-LXRα-LBD or pVP16-LXRβ-LBD plasmids (133 ng/300μl), described in [40], using the jetPrime transfection reagent (Polyplus). Six hours after transfection, we treated the cells with the appropriate compound for 40 hours. Luciferase activity was analyzed as described above and protein content spectrophotometrically using Coomassie Plus Protein assay reagent (Thermo Scientific).

#### Quantitative real-time-PCR

U937 cell line was differentiated in foam macrophages with phorbol 12-myristate 13-acetate (PMA) 10 ng/ml (Sigma) for 72 hours at 37 °C in RPMI 10% FBS. At day 3 T0901317 (10 μM) or different amount of PFM037 (50, 25 and 10 μM) were added for 16 hours. Total RNA was purified by TRIZOL (Invitrogen). Reverse transcription was performed by MLV-reverse transcriptase (Promega). QPCR was performed using SYBR Green Master Mix (Applied Biosystems) and real-time PCR (Viia 7 Real Time PCR System, Applied Biosystems). PCR reactions were done in triplicate. The comparative Ct method was used to quantify transcripts that were normalized for human GAPDH. We used the following primer pairs:

*GAPDH-F* 5’-ACA TCA TCC CTG CCT CTA CTG-3’; *GAPDH-R* 5’-ACC ACC TGG TGC TCA GTG TA-3’

*ABCA1-F* 5’-CCA GGC CAG TAC GGA ATT C-3’; *ABCA1-R* 5’-CCT CGC CAA ACC AGT AGG A-3’

*SREBP-1c-F* 5’*-*GGC GGG CGC AGA TC-3’; *SREBP-1c-R* 5’-TTG TTG ATA AGC TGA AGC ATG TCT-3’

*FAS-F* 5’-ACA GCG GGG AAT GGG TAC T-3’; *FAS-R* 5’-GAC TGG TAC AAC GAG CGG AT-3’

*SCD1-F* 5’-TTC AGA AAC ACA TGC TGA TCC TCA TAA TTC-3’; *SCD1-R* 5’*-*ATT AAG CAC CAC AGC ATA TCG CAA GAA AGT-3’

#### Blood plasma chemistry analysis

For all in vivo experiments mouse blood samples were collected via cardiac puncture and isolated plasma was analyzed by the COBAS c311 system (Roche) for plasma lipids.

#### Statistics

Sample size and power were calculated through formal analyses capable of defining a priori the extent of the underlying effect using the G*power 3.1 software. The number of animals per experimental group considers the need to reduce the use of animals to the minimum to obtain statistically significant results for the objectives of the study. Consistently with the underlying experimental design, we assumed a type I error equal to 5% (α = 0.05) and a power equal to 80% (1-β error prob = 0.80). In the various comparisons we chose to apply a Student’s t test evaluating the differences between two independent groups or an ANOVA test evaluating the differences among three or more groups. Data are expressed as mean ± s.d. and were analyzed for significance by ANOVA with Dunnet’s, Bonferroni’s or Tukey’s multiple comparison test, or by Student’s t test. Statistical comparison of overall survival was performed by the log-rank test. The analysis was performed with Prism software.

## Supplementary information

Scheme

Supplementary Figures 1-8

Supplementary Tables 1-5

aj-checklist

## Data Availability

RNA-seq data have been deposited in the GEO public repository and are accessible through the accession number GSE129746.
