# Supplementary figures and images for: Harnessing the reverse cholesterol transport pathway to favor differentiation of monocyte-derived APCs and antitumor responses
Source: Cell Death Dis. 2023 Feb 15;14(2):129. doi: 10.1038/s41419-023-05620-7 (PMC9932151; doi:10.1038/s41419-023-05620-7)

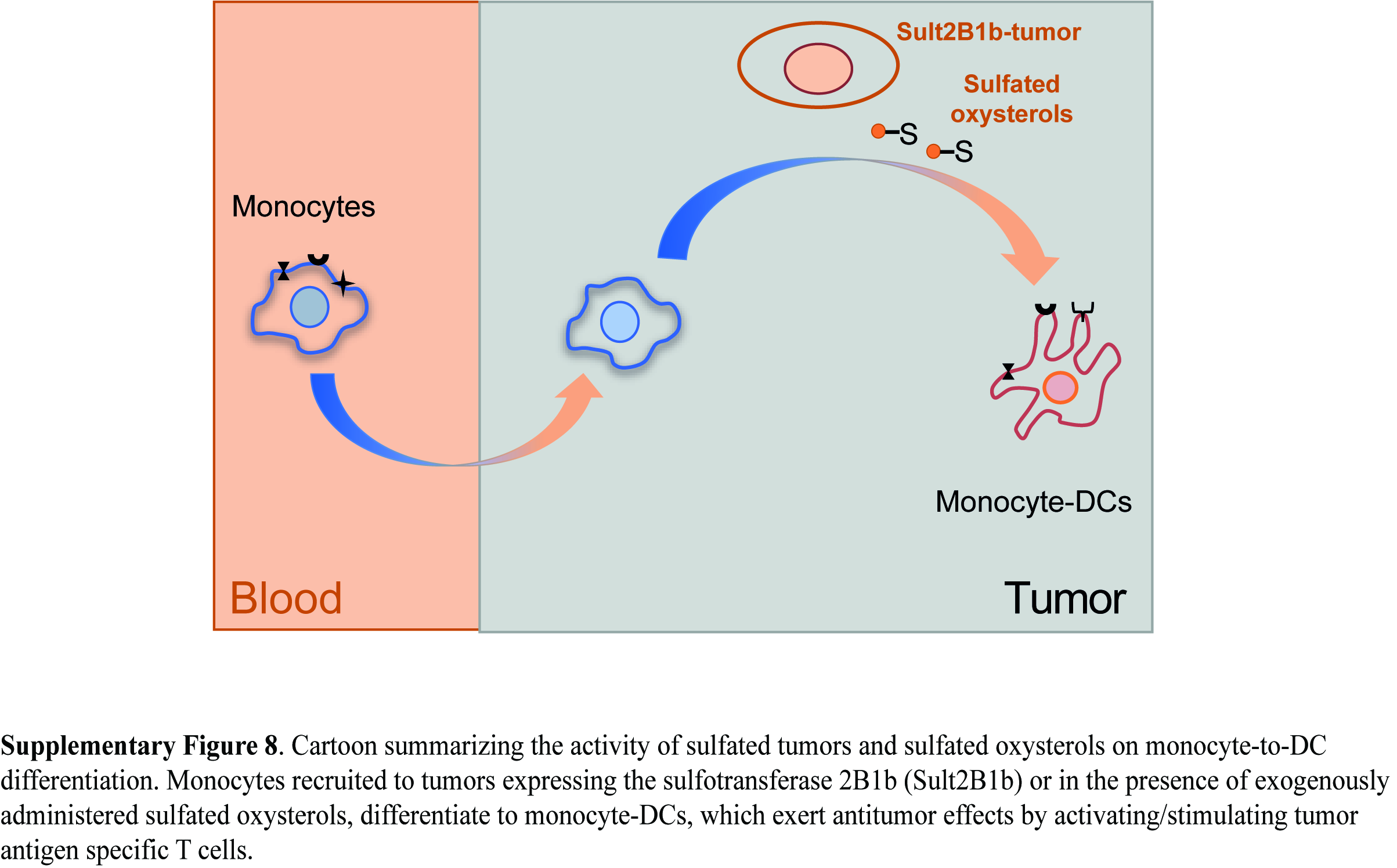

Supplement: Supplementary file 1 — Scheme [file 41419_2023_5620_MOESM1_ESM.tif]
