# Supplementary material for: Harnessing the reverse cholesterol transport pathway to favor differentiation of monocyte-derived APCs and antitumor responses
Source: Cell Death Dis. 2023 Feb 15;14(2):129. doi: 10.1038/s41419-023-05620-7 (PMC9932151; doi:10.1038/s41419-023-05620-7)
Supplement: Supplementary file 2 — Supplementary Figures 1-8 [file 41419_2023_5620_MOESM2_ESM.pdf]

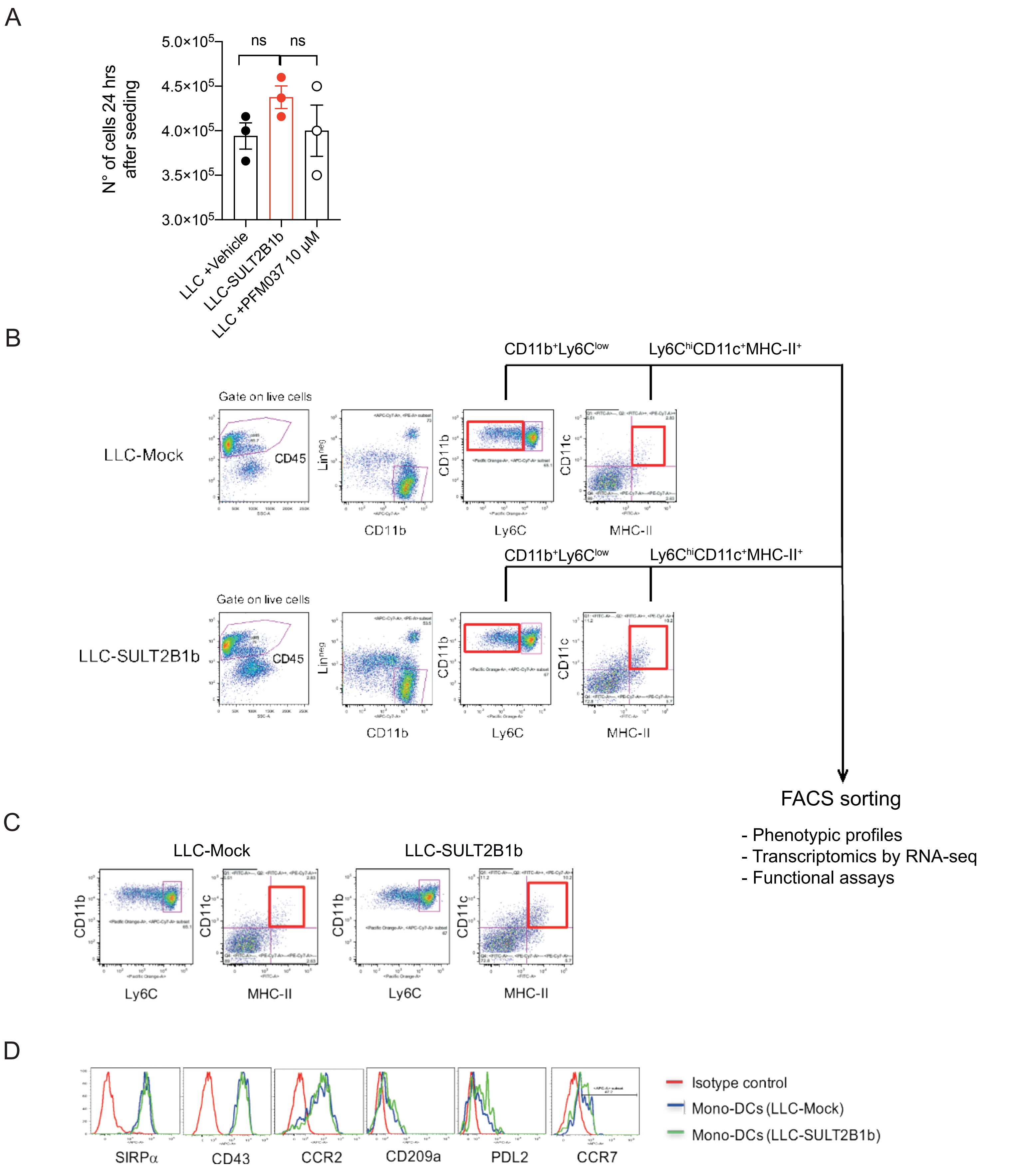

Supplementary Figure 1. (A) Number of LLC tumor cells, treated with vehicle, expressing Sult2B1b or treated with PFM037 (10 μM) for 24 hours. The expression of Sult2B1b or the addition of PFM037 do not alter the cell number. 3 x10^5 cells were plated in 6-well plates and counted the day after. Mean and s.d. of 3 experiments/group. *ns*, not significant (Anova). Gating strategy applied to characterize the tumor-infiltrating subsets of Ly6C$^+$ cells. (B, C) LLC-Mock and LLC-SULT2B1b tumors were digested and stained with a panel of mAbs against CD45, CD11b, Ly6C, CD11c and MHC-II molecules. Dead cells were excluded. Lineage negative cells were identified by the following mAbs: CD3, NK1.1, CD19, CD49b, Ly6G and Ter119. (D) Cells in the red box as in B were stained with mAbs against SIRPα, CD43, CCR2, CD209a, PDL2 and CCR7, which identify the mono-DCs. Ly6C^{high}CD11c$^+$MHC-II$^+$ and CD11b$^+$Ly6C^{low} cells were purified by FACS and analyzed by RNA-seq and functional assays.

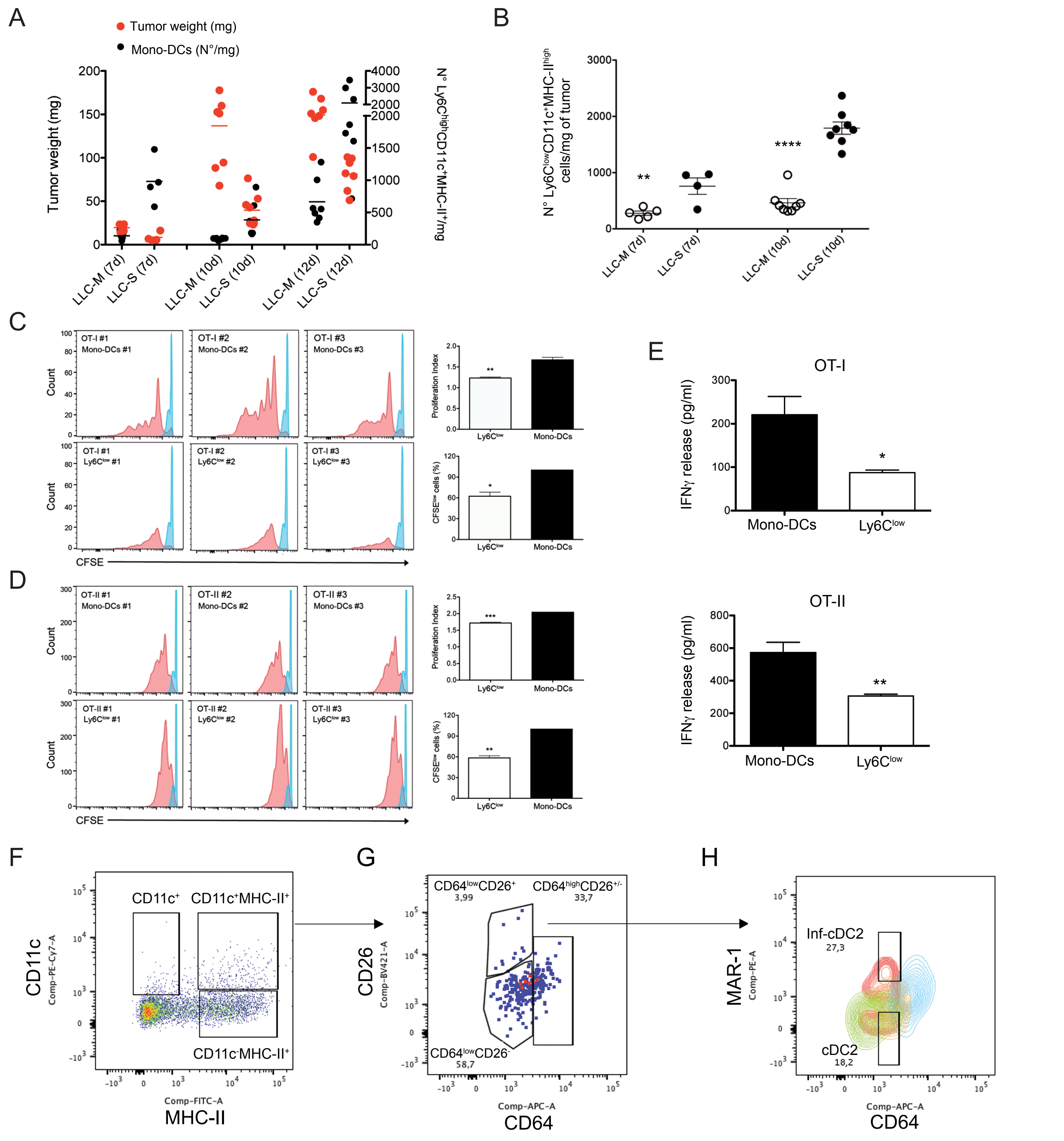

Supplementary Figure 2. (A) Tumor weight (mg) and absolute numbers of mono-DCs (Ly6C^{high}CD11c^{+}MHC-II^{+} cells)/mg of LLC-Mock (LLC-M) and LLC-SULT2B1b (LLC-S) tumors collected at different times. Day 7, mean and s.d. of 4-5 mice/group. Day 10, mean and s.d. of 8 mice/group. Day 12, mean and s.d. of 7 mice/group. (B) Number of Ly6C^{low}CD11c^{+}MHC-II^{+} cells/mg of LLC-Mock (LLC-M) and LLC-SULT2B1b (LLC-S) tumors collected at different different times. Day 7, mean and s.d. of 4-5 mice/group. Day 10, mean and s.d. of 8 mice/group. $**P < 0.01$; $****P < 0.0001$ (Student's t-test). (C-D) FACS analysis of OT-I (C) and OT-II (D) proliferation following in vivo priming with mono-DC or Ly6C^{low} cells isolated from LLC-SULT2B1b tumors and pulsed with MHC-I- and -II-restricted OVA peptides. Histograms represent the proliferation index of the percentage of OT-I (C) and OT-II (D) proliferation, evaluated as percentage of CFSE-diluted cells. Mean and s.d. of 3 experiments/group. $*P < 0.05$; $**P < 0.01$; $***P < 0.001$ (Student's t-test). (E) Supernatants from the experiments described in C and D were collected after 48 hours of co-culture and tested for IFN-γ release. Mean and s.d. of 3 experiments per group. $*P < 0.05$; $**P < 0.01$ (Student's t-test). (F-H) Tumors digested and stained with mAbs against CD45, CD11b, Ly6C, CD11c, MHC-II, CD26, CD64 and MAR-1 molecules. (G) Analysis for the expression of CD26 and CD64 molecules was performed on gated CD11c^{+}Ly6C^{high}MHC-II^{+} cells. (H) Analysis for the expression of MAR-1 marker was performed on gated CD64^{low}CD26^{+} cells.

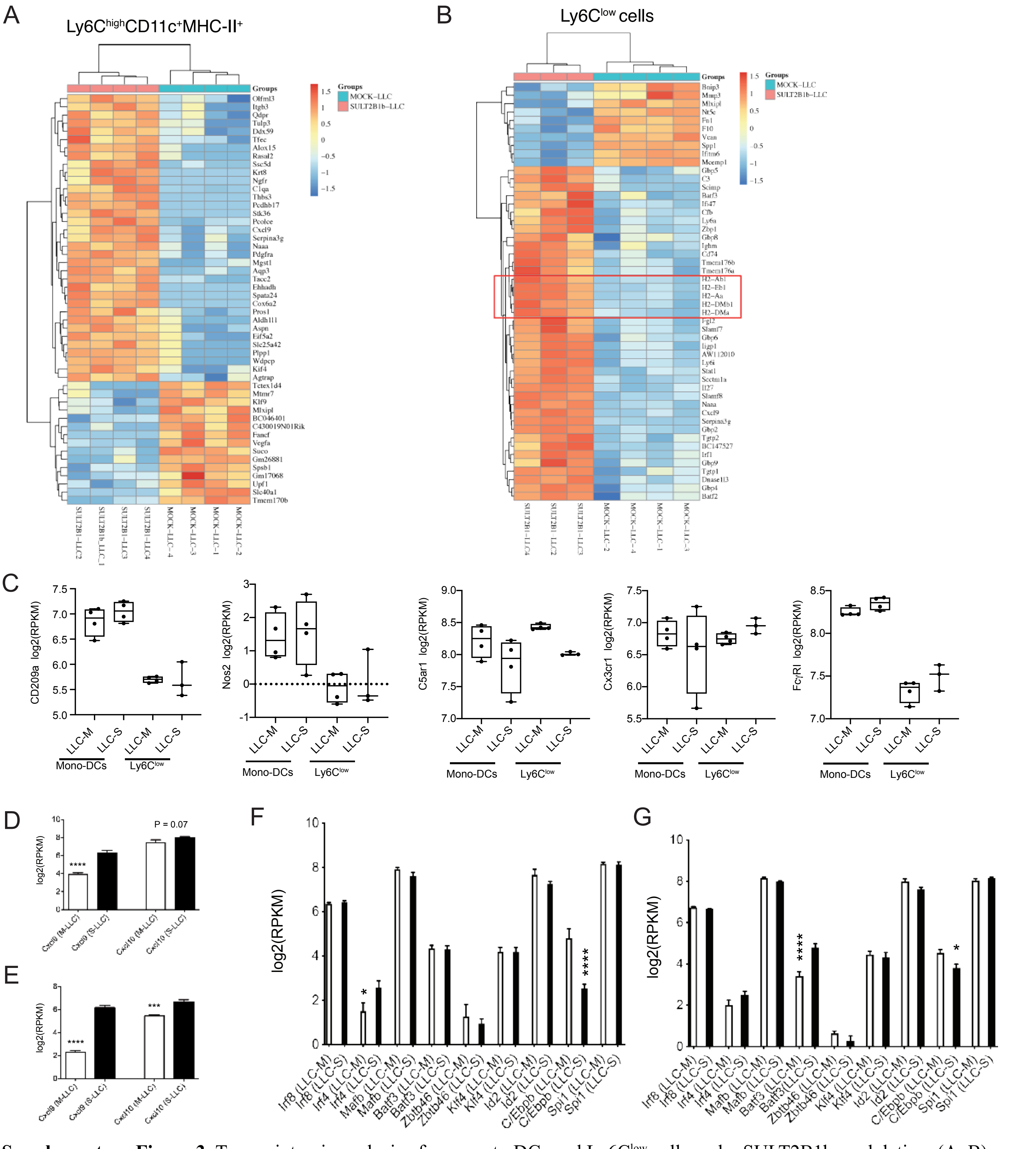

Supplementary Figure 3. Transcriptomic analysis of monocyte-DCs and Ly6C^low cells under SULT2B1b modulation. (A, B) Heatmap of 50 genes differentially expressed between monocyte-DCs (A) and Ly6C^low cells (B) isolated from LLC-Mock and LLC-SULT2B1b tumors. Expression levels are represented in the heatmap as log2(RPKM) transformed to zero mean and unit variance. Genes encoding MHC-II molecules differentially expressed by Ly6C^low cells from LLC-Mock and LLC-SULT2B1b tumors are highlighted (red square). (C) Boxplots showing the expression of *Cd209a*, *Nos2, C5ar1, Cx3cr1* and *FcγRI* transcripts in mono-DCs and Ly6C^low cells isolated from Mock-LLC (M-LLC) and SULT2B1b-LLC (S-LLC). Results are from RNA-seq data and are expressed as log2(RPKM). RPKM, Reads per Kilobase per Million. (D, E) Barplots representing the expression levels of *Cxcl9* and *Cxcl10* genes in mono-DCs (D) and Ly6C^low cells (D) from M-LLC and S-LLC. Results are from RNA-seq data and are expressed as log2(RPKM). *P* values refer to differential expression evaluated by limma. ****P* < 0.001; *****P* < 0.0001 (limma). (F, G) Barplots representing the expression levels of genes encoding distinct transcription factors in monocyte-DCs (F) and Ly6C^low cells (G) M-LLC and S-LLC. Results are from RNA-seq data and are expressed as log2(RPKM). *P* values refer to differential expression evaluated by limma. **P* < 0.05; *****P* < 0.0001 (limma).

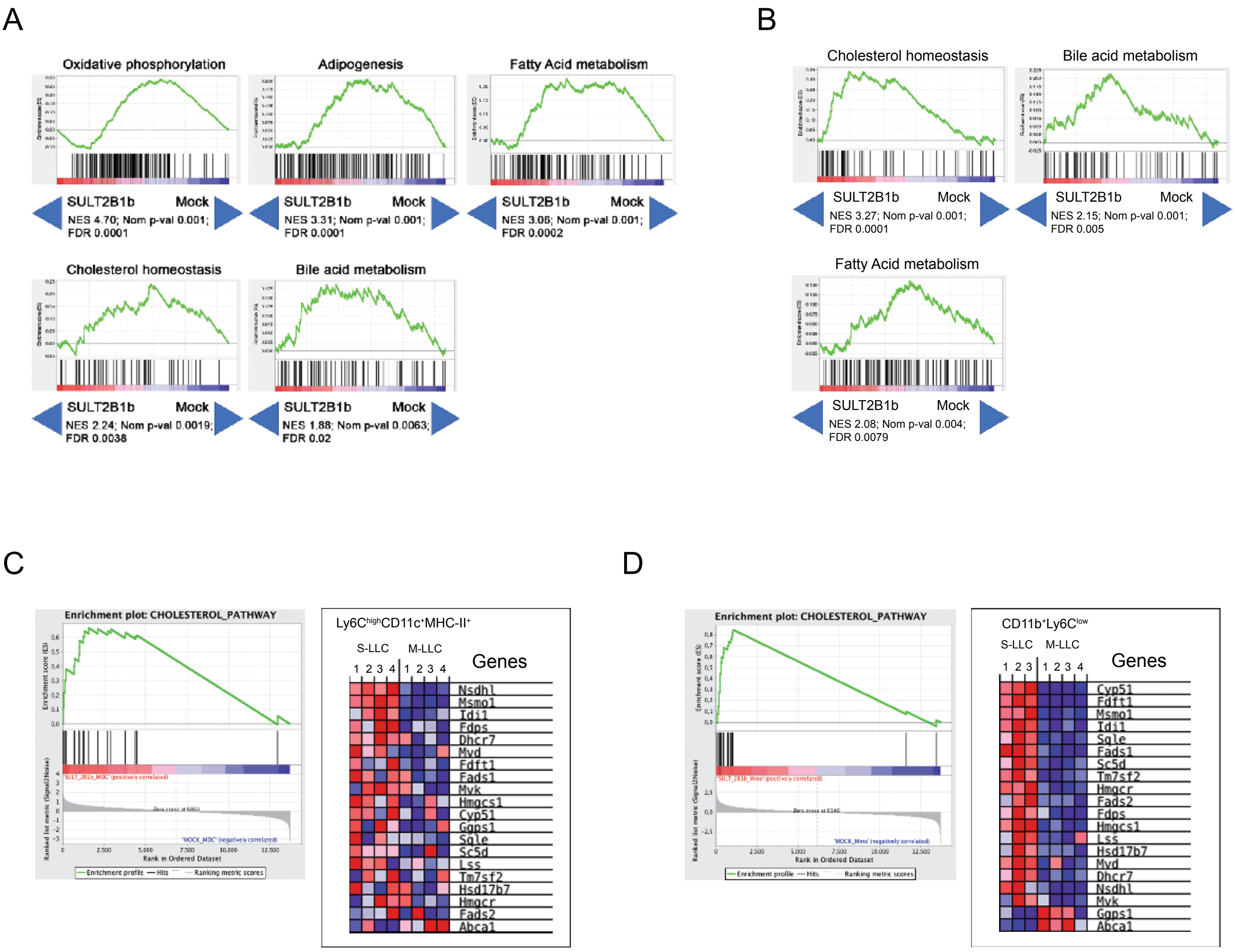

Supplementary Figure 4. GSEA of tumor-infiltrating Ly6C^{high}CD11c^{+}MHC-II^{+} and Ly6C^{low} cells under SULT2B1b modulation. (A) Gene Set Enrichment Analysis (GSEA) tracing for oxidative phosphorylation, adipogenesis, fatty acid metabolism, cholesterol homeostasis and bile acid metabolism of tumor infiltrating Ly6C^{high}CD11c^{+}MHC-II^{+} mono-DCs rom SULT2B1b- and Mock-LLC tumors. NES, normalized enrichment score; Nom p-val, Nominal p value; FDR, false discovery ratio. (B) GSEA tracing for cholesterol homeostasis, bile acid metabolism and fatty acid metabolism of tumor-infiltrating Ly6C^{low} cells from SULT2B1b- and Mock-LLC tumors. NES, normalized enrichment score; Nom p-val, Nominal p value; FDR, false discovery ratio. (C, D) GSEA using a signature of genes involved in LXR/cholesterol synthesis pathways, as reported in[32]. The gene set is significantly up-regulated in Ly6C^{high}CD11c^{+}MHC-II^{+} mono-DCs (C) and Ly6C^{low} cells (D) isolated from LLC-SULT2B1b tumors. Nominal p value < 1×10^{-4}. Abbreviations are as follows: S-LLC, LLC-SULT2B1b; M-LLC, LLC-Mock.

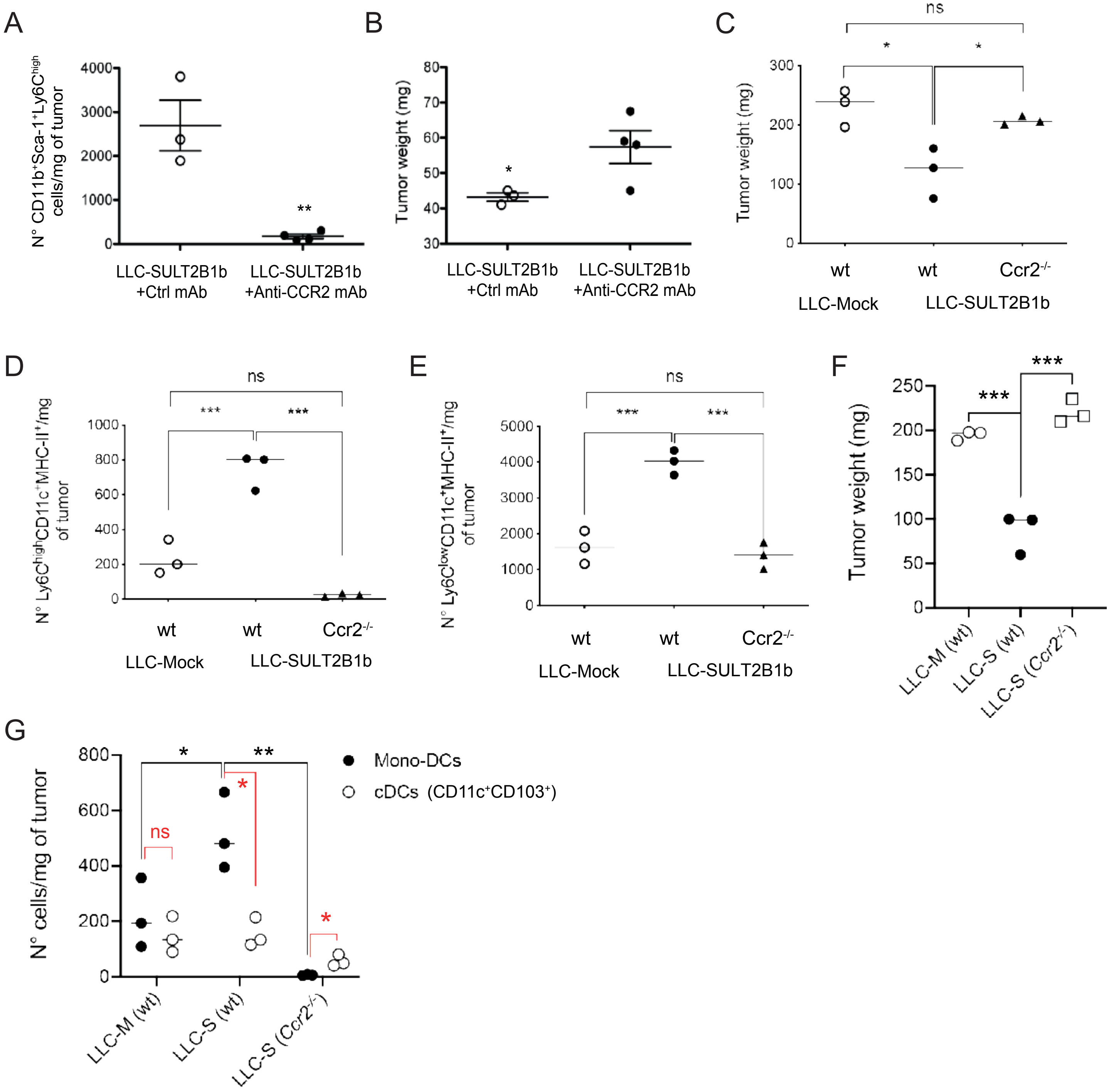

Supplementary Figure 5. Monocyte-DCs control tumor growth under SULT2B1b perturbation. (A, B) Mice bearing LLC-SULT2B1b tumors were treated with anti-CCR2 or ctrl mAbs for five consecutive days. At sacrifice, tumors were collected and analyzed for the content of tumor-infiltrating mono-DCs and for tumor weight. Absolute numbers of mono-DCs, evaluated as Ly6C^{high}CD11b^{+}Sca-1^{+} cells/mg of tumor (A) and tumor weight (B) between the two groups of treatment. Results are presented as mean ± s.d. of 3-4 mice/group; *P < 0.05; **P < 0.01 (Student's t-test). (C-E) *Ccr2*$^{-/-}$ and wild type mice were infused with LLC-Mock and LLC-SULT2B1b tumors. At sacrifice, tumors were collected and analyzed for the content of monocyte-DCs, Ly6C^{low}CD11c^{+}MHC-II^{+} cells and tumor weight. Tumor weights (C), absolute numbers of monocyte-DCs (D) and Ly6C^{low}CD11c^{+}MHC-II^{+} cells/mg of tumor (E) between the groups of treatment. Mean and s.d. of 3 mice/group. ns, not significant; *P < 0.05; ***P < 0.001 (ANOVA). (F-G) *Ccr2*$^{-/-}$ and wild type mice were infused with LLC-Mock and LLC-SULT2B1b tumors. At sacrifice, tumors were collected and analyzed as described above. (F) Tumor weights between the groups of treatment. Mean and s.d. of 3 mice/group. ***p < 0.001 (ANOVA). (G) Absolute numbers of monocyte-DCs and (CD103^{+}CD11c^{+}) cDCs/mg of tumor. Mean and s.d. of 3 mice/group. ns, not significant; *P < 0.05; **P < 0.01 (ANOVA). cDCs were not significantly different among the three groups of tumors (ANOVA).

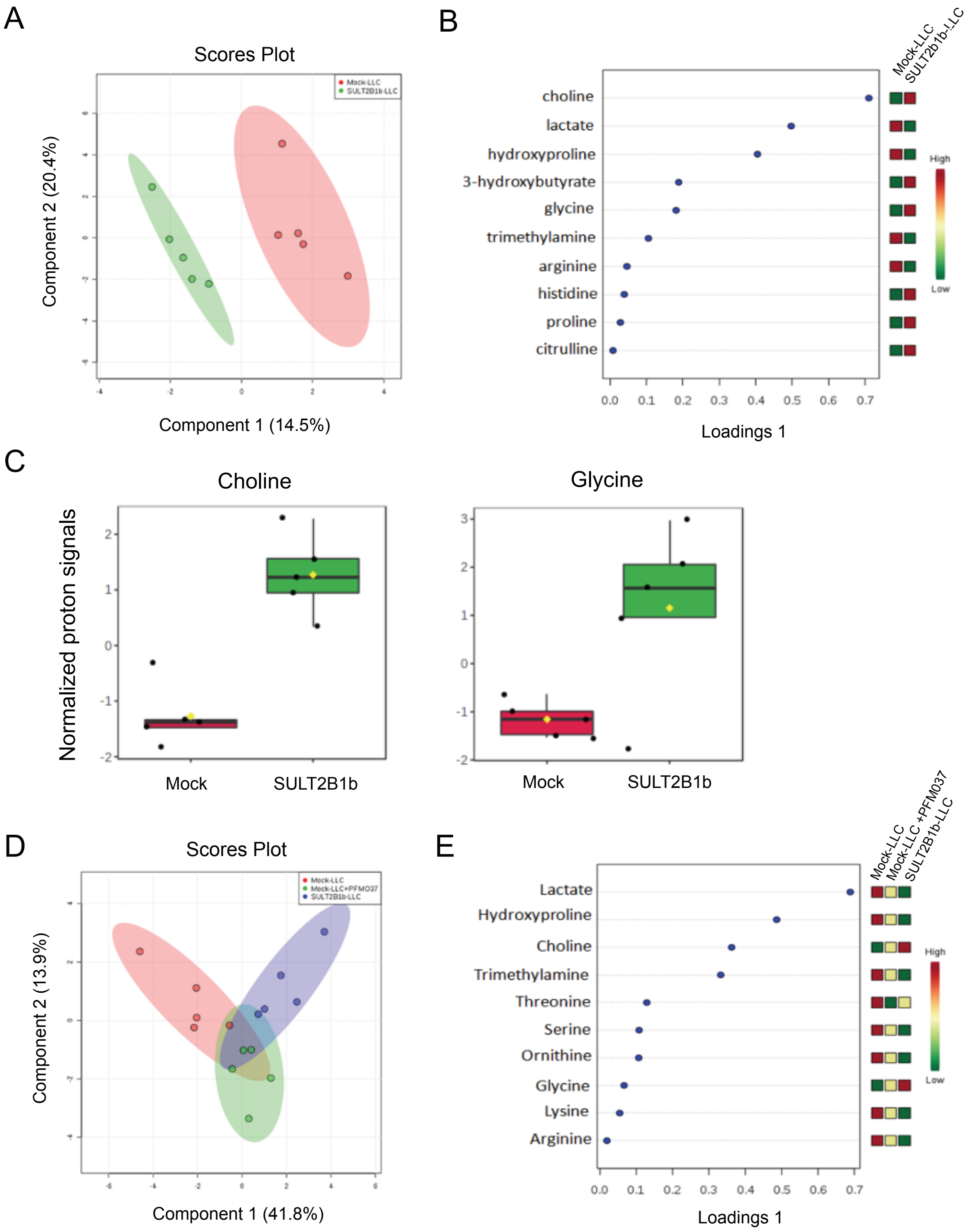

Supplementary Figure 6. Un-targeted metabolomics analysis of LLC-Mock and LLC-SULT2B1b, LLC from mice treated with vehicle or PFM037. (A) Score plot by Partial least squares-discriminant analysis (PLS-DA) related to the polar fraction of Mock and SULT2B1b-LLC tumors (n = 5 tumors/group). (B) Variable importance in projection (VIP) plot showing the top 10 NMR signals resulted more statistically different in the polar fractions of Mock and SULT2B1b-LLC tumors (n = 5tumors/group). (C) Box plots showing the levels of choline and glycine, evaluated as normalized proton signals, differentially expressed by Mock- and SULT2B1b-LLC tumors (n = 5 tumors/group). (D) Score plot by Partial least squares-discriminant analysis (PLS-DA) related to the polar fractions of Mock-LLC, SULT2B1b-LLC and Mock-LLC treated with PFM037 tumors (n = 5). (E) Variable importance in projection (VIP) plot showing the top 10 NMR signals resulted more statistically different in the polar fractions of Mock-LLC, SULT2B1b-LLC and Mock-LLC treated with PFM037 tumors (n = 5).

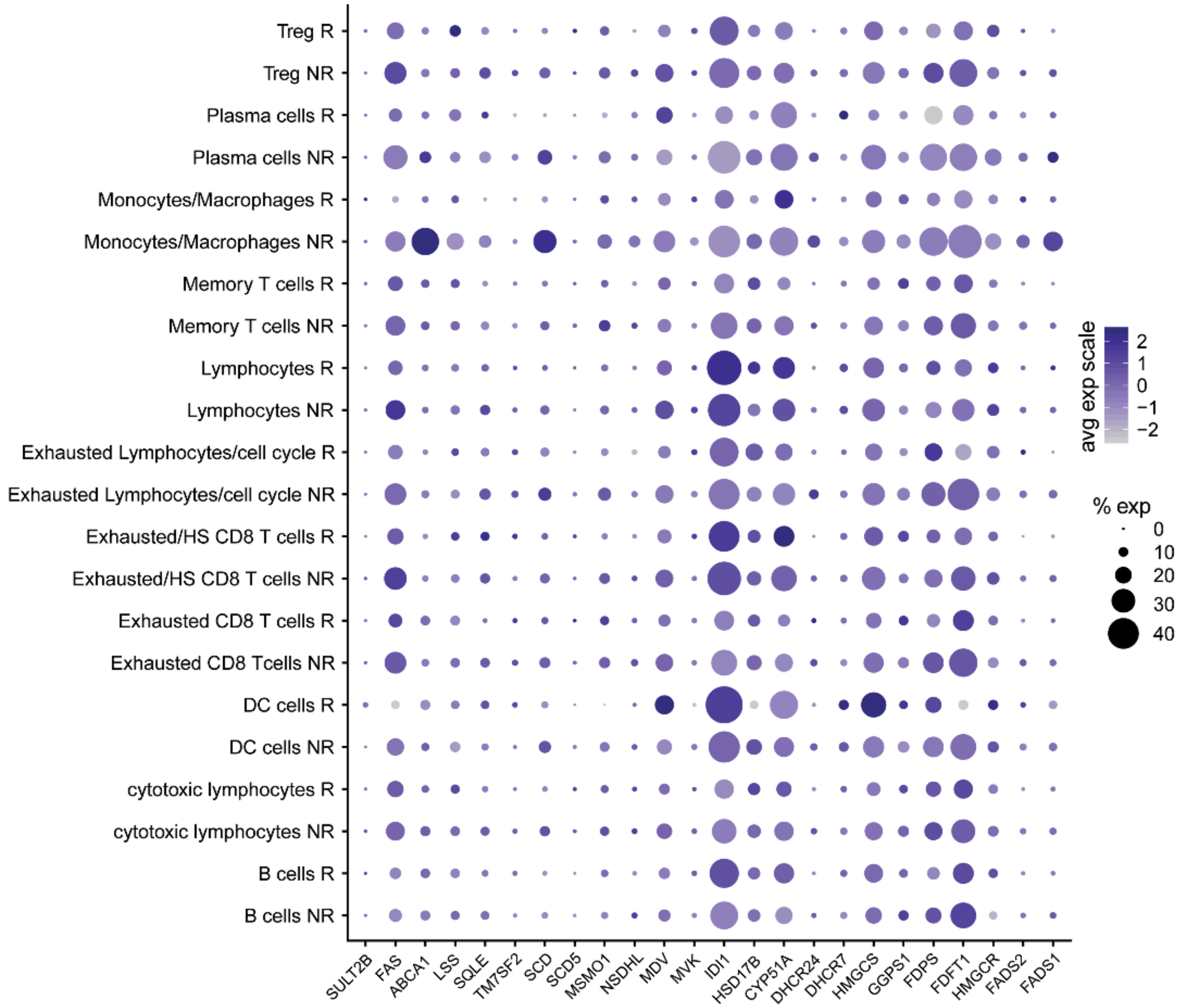

Supplementary Figure 7. Dot Plots of LXR/cholesterol synthesis signature genes in all melanoma-infiltrating CD45$^+$ cells profiled, divided in Responder (R) and Non Responder (NR) patient-cells[44]. The intensity of color in each dot represents the expression value. The size of each dot is related to the percentage of cells expressing the gene in the population of interest.

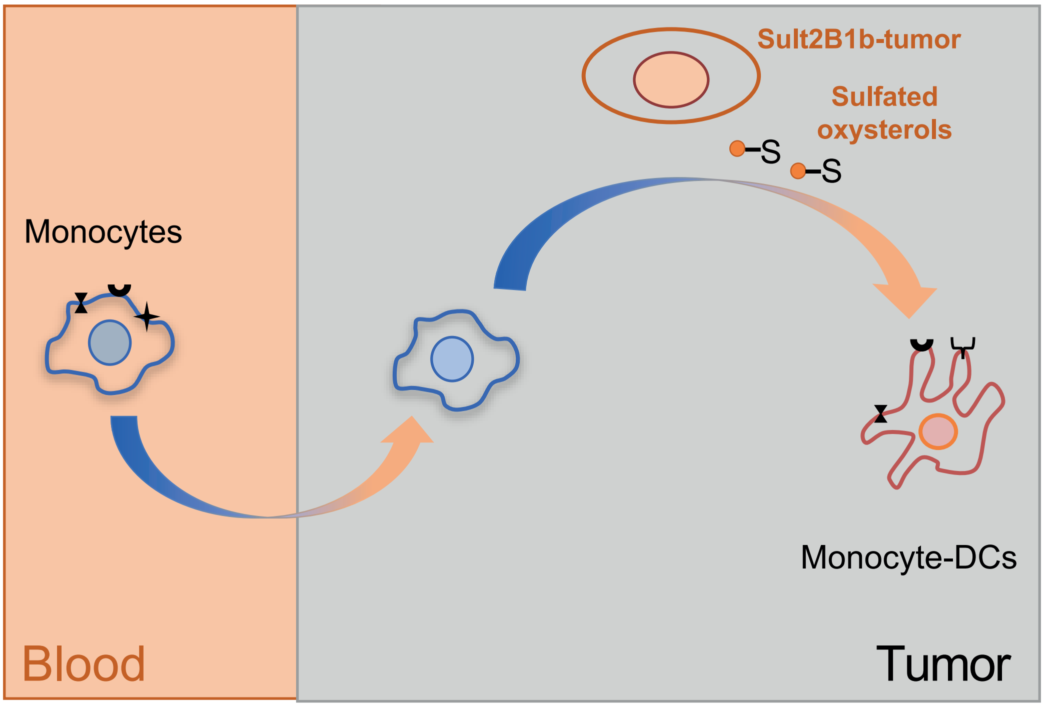

Supplementary Figure 8. Cartoon summarizing the activity of sulfated tumors and sulfated oxysterols on monocyte-to-DC differentiation. Monocytes recruited to tumors expressing the sulfotransferase 2B1b (Sult2B1b) or in the presence of exogenously administered sulfated oxysterols, differentiate to monocyte-DCs, which exert antitumor effects by activating/stimulating tumor antigen specific T cells.
