# Supplementary material for: Harnessing the reverse cholesterol transport pathway to favor differentiation of monocyte-derived APCs and antitumor responses
Source: Cell Death Dis. 2023 Feb 15;14(2):129. doi: 10.1038/s41419-023-05620-7 (PMC9932151; doi:10.1038/s41419-023-05620-7)
Supplement: Supplementary file 3 — Supplementary Tables 1-5 [file 41419_2023_5620_MOESM3_ESM.pdf]

Supplemental Table 1. GSEA up-regulated pathway Mono-DCs

NAME	GS<br>follow link to MSigDB	S DETAIL	SIZE	ES	NES	NOM p-val	FDR q-val	FWER p-val	RANK AT MAX	LEADING EDGE
HALLMARK	HALLMARK_OXIDATIVE_PHOSPHORYLATION	Details ...	192	0,290913	4,708063	0	0	0	8094	tags=89%, list=60%, signal=222%
HALLMARK	HALLMARK_MYC_TARGETS_V1	Details ...	195	0,220967	3,614919	0	0	0	8079	tags=82%, list=60%, signal=204%
HALLMARK	HALLMARK_EPITHELIAL_MESENCHYMAL_TRA	Details ...	135	0,257398	3,496142	0	0	0	2839	tags=47%, list=21%, signal=59%
HALLMARK	HALLMARK_DNA_REPAIR	Details ...	141	0,241783	3,362606	0	0	0	7535	tags=80%, list=56%, signal=181%
HALLMARK	HALLMARK_ADIPOGENESIS	Details ...	180	0,206448	3,31902	0	0	0	7248	tags=74%, list=54%, signal=160%
HALLMARK	HALLMARK_FATTY_ACID_METABOLISM	Details ...	125	0,22891	3,060456	0,001941748	2,67E-04	0,001	5860	tags=66%, list=44%, signal=117%
HALLMARK	HALLMARK_INTERFERON_GAMMA_RESPONS	Details ...	173	0,203444	3,006807	0	2,29E-04	0,001	8155	tags=81%, list=61%, signal=204%
HALLMARK	HALLMARK_ESTROGEN_RESPONSE_LATE	Details ...	132	0,177459	2,404261	0,002008032	0,001599583	0,008	3229	tags=42%, list=24%, signal=54%
HALLMARK	HALLMARK_INTERFERON_ALPHA_RESPONSE	Details ...	88	0,215193	2,360275	0	0,001980582	0,011	8101	tags=82%, list=60%, signal=205%
HALLMARK	HALLMARK_XENOBIOTIC_METABOLISM	Details ...	142	0,170769	2,344286	0,004048583	0,001782524	0,011	5475	tags=58%, list=41%, signal=97%
HALLMARK	HALLMARK_PEROXISOME	Details ...	79	0,230983	2,340413	0	0,001750346	0,012	4387	tags=56%, list=33%, signal=82%
HALLMARK	HALLMARK_MTORC1_SIGNALING	Details ...	192	0,142905	2,305663	0,002012072	0,002074559	0,016	9142	tags=82%, list=68%, signal=255%
HALLMARK	HALLMARK_CHOLESTEROL_HOMEOSTASIS	Details ...	64	0,239312	2,24177	0,001923077	0,00384678	0,03	7279	tags=78%, list=54%, signal=170%
HALLMARK	HALLMARK_UNFOLDED_PROTEIN_RESPONSI	Details ...	105	0,187068	2,223052	0,004106776	0,00357201	0,03	9384	tags=89%, list=70%, signal=293%
HALLMARK	HALLMARK_E2F_TARGETS	Details ...	198	0,135425	2,215705	0	0,003440542	0,031	6944	tags=65%, list=52%, signal=133%
HALLMARK	HALLMARK_MYOGENESIS	Details ...	119	0,172158	2,197394	0,006185567	0,003605229	0,035	1767	tags=30%, list=13%, signal=35%
HALLMARK	HALLMARK_ALLOGRAFT_REJECTION	Details ...	148	0,134078	1,898751	0,012048192	0,022078617	0,209	8547	tags=77%, list=64%, signal=210%
HALLMARK	HALLMARK_BILE_ACID_METABOLISM	Details ...	72	0,188558	1,886446	0,006302521	0,022716086	0,225	4746	tags=54%, list=35%, signal=83%
HALLMARK	HALLMARK_GLYCOLYSIS	Details ...	165	0,123492	1,860182	0,014957265	0,0247271	0,25	8763	tags=78%, list=65%, signal=221%
HALLMARK	HALLMARK_COAGULATION	Details ...	90	0,162594	1,843649	0,008032128	0,025886329	0,272	6026	tags=61%, list=45%, signal=110%
HALLMARK	HALLMARK_UV_RESPONSE_DN		123	0,132099	1,731917	0,024809161	0,044129826	0,441	1296	tags=23%, list=10%, signal=25%
HALLMARK	HALLMARK_ANGIOGENESIS		29	0,264212	1,713817	0,027613413	0,046008147	0,47	4323	tags=59%, list=32%, signal=86%
HALLMARK	HALLMARK_INFLAMMATORY_RESPONSE		161	0,112244	1,696394	0,025210084	0,048508123	0,507	10502	tags=89%, list=78%, signal=408%
HALLMARK	HALLMARK_UV_RESPONSE_UP		125	0,122825	1,540513	0,056133058	0,09553659	0,788	10486	tags=90%, list=78%, signal=411%
HALLMARK	HALLMARK_APOPTOSIS		139	0,110016	1,51031	0,05462185	0,106214486	0,828	9630	tags=83%, list=72%, signal=291%
HALLMARK	HALLMARK_ESTROGEN_RESPONSE_EARLY		134	0,1109	1,508885	0,05019305	0,103222445	0,83	3229	tags=35%, list=24%, signal=46%
HALLMARK	HALLMARK_IL6_JAK_STAT3_SIGNALING		76	0,143132	1,47678	0,07587548	0,11192989	0,868	9556	tags=86%, list=71%, signal=296%
HALLMARK	HALLMARK_COMPLEMENT		148	0,103066	1,4639	0,0781893	0,11432432	0,885	9411	tags=80%, list=70%, signal=267%
HALLMARK	HALLMARK_PROTEIN_SECRETION		90	0,129153	1,413877	0,11157025	0,1374338	0,934	9897	tags=87%, list=74%, signal=329%
HALLMARK	HALLMARK_MITOTIC_SPINDLE		193	0,087859	1,407176	0,10465116	0,13607894	0,936	10993	tags=91%, list=82%, signal=497%
HALLMARK	HALLMARK_REACTIVE_OXIGEN_SPECIES_PATHWAY		44	0,178283	1,401605	0,10852713	0,13478337	0,937	9194	tags=86%, list=69%, signal=274%
HALLMARK	HALLMARK_SPERMATOGENESIS		69	0,144071	1,399157	0,118236475	0,13206542	0,939	2546	tags=33%, list=19%, signal=41%
HALLMARK	HALLMARK_KRAS_SIGNALING_DN		63	0,139026	1,283617	0,1594203	0,2062612	0,986	3038	tags=37%, list=23%, signal=47%
HALLMARK	HALLMARK_APICAL_SURFACE		23	0,220221	1,259991	0,18448637	0,21938212	0,992	10457	tags=100%, list=78%, signal=454%
HALLMARK	HALLMARK_G2M_CHECKPOINT		190	0,073115	1,154092	0,27875245	0,3097754	0,998	1996	tags=22%, list=15%, signal=26%
HALLMARK	HALLMARK_ANDROGEN_RESPONSE		84	0,099423	1,088798	0,34268537	0,37140992	0,999	10005	tags=85%, list=75%, signal=331%
HALLMARK	HALLMARK_HEDGEHOG_SIGNALING		22	0,175137	0,984522	0,4653846	0,4938427	1	3139	tags=41%, list=23%, signal=53%
HALLMARK	HALLMARK_PANCREAS_BETA_CELLS		14	0,221193	0,984357	0,4573991	0,48095262	1	8527	tags=86%, list=64%, signal=235%
HALLMARK	HALLMARK_PI3K_AKT_MTOR_SIGNALING		91	0,085358	0,948228	0,50884086	0,5155349	1	11531	tags=95%, list=86%, signal=671%
HALLMARK	HALLMARK_APICAL_JUNCTION		126	0,065806	0,847676	0,6205451	0,64593214	1	8381	tags=69%, list=63%, signal=183%

Supplemental Table 2. GSEA up-regulated pathway Ly6Clow cells

NAME	GS<br>follow link to MSigDB	GS DETAILS	SIZE	ES	NES	NOM p-val	FDR q-val	FWER p-val	RANK AT MAX	LEADING EDGE
HALLMARK_	HALLMARK_INTERFERON_GAMMA_RESPON	Details ...	173	0,38793	6,066713	0	0	0	5094	tags=76%, list=38%, signal=121%
HALLMARK_	HALLMARK_INTERFERON_ALPHA_RESPON	Details ...	88	0,466978	5,153086	0	0	0	3834	tags=75%, list=29%, signal=104%
HALLMARK_	HALLMARK_E2F_TARGETS	Details ...	198	0,246279	4,016788	0	0	0	6834	tags=75%, list=51%, signal=151%
HALLMARK_	HALLMARK_INFLAMMATORY_RESPONSE	Details ...	161	0,247853	3,676484	0	0	0	2961	tags=47%, list=22%, signal=59%
HALLMARK_	HALLMARK_CHOLESTEROL_HOMEOSTASIS	Details ...	64	0,340451	3,277226	0	0	0	2369	tags=52%, list=18%, signal=62%
HALLMARK_	HALLMARK_G2M_CHECKPOINT	Details ...	190	0,192797	3,103175	0	0	0	4577	tags=53%, list=34%, signal=80%
HALLMARK_	HALLMARK_TNFA_SIGNALING_VIA_NFKB	Details ...	186	0,192207	3,026055	0	0	0	4449	tags=52%, list=33%, signal=77%
HALLMARK_	HALLMARK_MYC_TARGETS_V1	Details ...	195	0,178507	2,908859	0	0	0	10221	tags=94%, list=76%, signal=389%
HALLMARK_	HALLMARK_IL6_JAK_STAT3_SIGNALING	Details ...	76	0,228177	2,339181	0	0,0011443	0,008	6835	tags=74%, list=51%, signal=149%
HALLMARK_	HALLMARK_ESTROGEN_RESPONSE_LATE	Details ...	132	0,169963	2,298017	0,0020534	0,0014185	0,011	5258	tags=56%, list=39%, signal=91%
HALLMARK_	HALLMARK_BILE_ACID_METABOLISM	Details ...	72	0,214488	2,154799	0	0,0050317	0,043	5145	tags=60%, list=38%, signal=96%
HALLMARK_	HALLMARK_FATTY_ACID_METABOLISM	Details ...	125	0,159726	2,083002	0,0040486	0,0079668	0,075	6886	tags=67%, list=51%, signal=137%
HALLMARK_	HALLMARK_ALLOGRAFT_REJECTION	Details ...	148	0,14816	2,079041	0,0041408	0,0074524	0,076	3107	tags=38%, list=23%, signal=49%
HALLMARK_	HALLMARK_DNA_REPAIR	Details ...	141	0,146363	1,988095	0,0021097	0,0127134	0,132	10607	tags=94%, list=79%, signal=444%
HALLMARK_	HALLMARK_IL2_STAT5_SIGNALING	Details ...	169	0,125655	1,90822	0,006383	0,019553	0,211	3095	tags=36%, list=23%, signal=46%
HALLMARK_	HALLMARK_KRAS_SIGNALING_DN	Details ...	63	0,202718	1,889664	0,0060362	0,0199278	0,225	4316	tags=52%, list=32%, signal=77%
HALLMARK_	HALLMARK_COMPLEMENT	Details ...	148	0,134578	1,883834	0,0114504	0,0193875	0,233	5642	tags=55%, list=42%, signal=95%
HALLMARK_	HALLMARK_UV_RESPONSE_UP	Details ...	125	0,141452	1,876599	0,0165975	0,0191243	0,243	3697	tags=42%, list=28%, signal=57%
HALLMARK_	HALLMARK_MYC_TARGETS_V2	Details ...	56	0,189687	1,671399	0,03	0,0512763	0,532	9675	tags=91%, list=72%, signal=326%
HALLMARK_	HALLMARK_KRAS_SIGNALING_UP	Details ...	142	0,118514	1,663568	0,0219124	0,0499616	0,539	1920	tags=26%, list=14%, signal=30%
HALLMARK_	HALLMARK_PEROXISOME		79	0,130284	1,370081	0,1120332	0,1859795	0,961	9971	tags=87%, list=74%, signal=339%
HALLMARK_	HALLMARK_ADIPOGENESIS		180	0,084823	1,355823	0,1234818	0,1884226	0,969	10197	tags=84%, list=76%, signal=348%
HALLMARK_	HALLMARK_P53_PATHWAY		178	0,084158	1,326759	0,1535509	0,2030375	0,98	3555	tags=35%, list=27%, signal=47%
HALLMARK_	HALLMARK_REACTIVE_OXIGEN_SPECIES_PATHWAY		44	0,151891	1,143076	0,2865854	0,3770714	0,999	8328	tags=77%, list=62%, signal=203%
HALLMARK_	HALLMARK_PI3K_AKT_MTOR_SIGNALING		91	0,101859	1,128695	0,2831325	0,3812877	0,999	9249	tags=79%, list=69%, signal=253%
HALLMARK_	HALLMARK_MITOTIC_SPINDLE		193	0,070251	1,114959	0,3203125	0,3819444	0,999	11434	tags=92%, list=85%, signal=618%
HALLMARK_	HALLMARK_SPERMATOGENESIS		69	0,113035	1,112421	0,300578	0,3708077	0,999	3737	tags=39%, list=28%, signal=54%
HALLMARK_	HALLMARK_XENOBIOTIC_METABOLISM		142	0,079057	1,084857	0,3477407	0,3917155	0,999	8013	tags=68%, list=60%, signal=166%
HALLMARK_	HALLMARK_APOPTOSIS		139	0,068515	0,914933	0,5641548	0,6050655	1	2369	tags=24%, list=18%, signal=29%
HALLMARK_	HALLMARK_NOTCH_SIGNALING		25	0,150747	0,893843	0,5653061	0,6151704	1	8706	tags=80%, list=65%, signal=228%
HALLMARK_	HALLMARK_ANDROGEN_RESPONSE		84	0,071053	0,766123	0,7712551	0,7862689	1	3840	tags=36%, list=29%, signal=50%
HALLMARK_	HALLMARK_MTORC1_SIGNALING		192	0,045921	0,732261	0,7992126	0,8072394	1	9446	tags=75%, list=70%, signal=250%

Supplemental Table 3. Tumor-infiltrating monocytes/macrophages NR vs R

	Avg. logFC	Pct. 1 (%)	Pct. 2 (%)	P val adj
ABCA1	1.521254	35.1	5.9	4.29×10^{-8}
SCD	1.803138	29.2	5.2	2.93×10^{-5}

Supplemental Table 4. Tumor-infiltrating DCs R vs NR

	Avg. logFC	Pct. 1 (%)	Pct. 2 (%)	P val adj
HMGCS1	2.377323	31.9	26.3	1
MVD	1.254463	23.4	17.72	1

Supplemental Table 5. Tumor-infiltrating monocytes/macrophages NR vs all

	Avg. logFC	Pct. 1 (%)	Pct. 2 (%)	*P* val adj
ABCA1	1.404119	35.1	7.8	5.79×10^{-212}
SCD	0.68751	29.2	8.8	1.23×10^{-105}
